# Synergistic Effects in N,O‐Comodified Carbon Nanotubes Boost Highly Selective Electrochemical Oxygen Reduction to H_2_O_2_

**DOI:** 10.1002/advs.202201421

**Published:** 2022-07-28

**Authors:** Shuhui Xu, Ruihu Lu, Kai Sun, Jialun Tang, Yaping Cen, Liang Luo, Ziyun Wang, Shubo Tian, Xiaoming Sun

**Affiliations:** ^1^ State Key Laboratory of Chemical Resource Engineering College of Chemistry Beijing University of Chemical Technology Beijing 100029 China; ^2^ School of Chemical Sciences The University of Auckland Auckland 1010 New Zealand; ^3^ State Power Investment Corporation hydrogen energy Co., Ltd. Beijing 100029 China

**Keywords:** asymmetric wettability, DFT, hydrogen peroxide, modified carbon nanotubes, oxygen reduction reaction

## Abstract

Electrochemical 2‐electron oxygen reduction reaction (ORR) is a promising route for renewable and on‐site H_2_O_2_ production. Oxygen‐rich carbon nanotubes have been demonstrated their high selectivity (≈80%), yet tailoring the composition and structure of carbon nanotubes to further enhance the selectivity and widen working voltage range remains a challenge. Herein, combining formamide condensation coating and mild temperature calcination, a nitrogen and oxygen comodified carbon nanotubes (N,O‐CNTs) electrocatalyst is synthesized, which shows excellent selective (>95%) H_2_O_2_ selectivity in a wide voltage range (from 0 to 0.65 V versus reversible hydrogen electrode). It is significantly superior to the corresponding selectivity values of CNTs (≈50% in 0–0.65 V vs RHE) and O‐CNTs (≈80% in 0.3–0.65 V vs RHE). Density functional theory calculations revealed that the C neighbouring to N is the active site. Introducing O‐related species can strengthen the adsorption of intermediates *OOH, while N‐doping can weaken the adsorption of in situ generated *O and optimize the *OOH adsorption energy, thus improving the 2‐electron pathway. With optimized N,O‐CNTs catalysts, a Janus electrode is designed by adjusting the asymmetric wettability to achieve H_2_O_2_ productivity of 264.8 mol kg_cat_^–1^ h^–1^.

## Graphical abstract

Using the double‐heteroatom comodified strategy, this work design a N,O‐comodified carbon nanotubes (N,O‐CNTs) electrocatalyst. The O and N atoms can regulate the adsorption energy of *OOH and *O, and the selectivity for H_2_O_2_ reaches more than 95%. Moreover, a Janus electrode is designed using the catalyst to achieve H_2_O_2_ productivity of 264.8 mol kg_cat_^−1^ h^−1^.

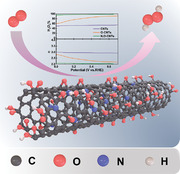

## 1 Introduction

Hydrogen peroxide (H_2_O_2_) is a valuable chemical widely used in chemical oxidation, textile, paper manufacturing, and other industries.^[^ ^1^ ^]^ It turns more critical, especially in the medical area at present rampant epidemic condition.^[^ ^2^ ^]^ Nowadays, the production of H_2_O_2_ is mainly based on the well‐established anthraquinone oxidation method, which could produce concentrated H_2_O_2_.^[^ ^3^ ^]^ However, the storage and transport of bulk high concentration H_2_O_2_ are hazardous and expensive.^[^ ^4^ ^]^ Electrochemical oxygen reduction reaction (ORR) through the 2‐electron pathway is a green, safe, and distributed method that only uses oxygen in the air to produce H_2_O_2_ in situ, without the transportation process.^[^ ^5^ ^]^ However, this method critically requires developing efficient and stable electrocatalysts selective toward the 2‐electron ORR, even at fluctuated voltages.^[^ ^6^ ^]^

During the exploration for optimized catalysts, noble metal‐based catalysts, such as Pt, Au, Pd, and Pt–Hg alloys, first demonstrated their high 2‐electron ORR performance,^[^ ^7^ ^]^ but their high cost and scarcity limit their large‐scale application.^[^ ^8^ ^]^ Atomically dispersed nonprecious M‐N/C catalysts (M for transition metal atoms) also show high activity in 2‐electron ORR performance.^[^ ^9^ ^]^ However, there is a risk of metal leaching, and many organic compound precursors for the synthesis of M‐N/C are environment‐unfriendly.^[^ ^10^ ^]^ Mechanistically, the control of the selectivity of the two‐/four‐electron reaction lies mainly in the adsorbed intermediate product *OOH.^[^ ^11^ ^]^ The strong adsorption for *OOH tends to the four‐electron process, while too weak adsorption for *OOH could reduce the overall activity.^[^ ^12^ ^]^ Carbon‐based metal‐free catalysts are abundant and flexible in structure, making them ideal substitutes for the electrochemical synthesis of H_2_O_2_.^[^ ^13^ ^]^ Current studies showed that heteroatoms doping, such as O, N, F, S, B, etc.,^[^ ^14^ ^]^ is an effective strategy to engineer the electronic structure and optimize the adsorption energy of *OOH, which showed promising performance.^[^ ^13a^ ^]^ For example, Iglesias et al. proposed a graphitized N‐doped carbon nanoangle (CNHs) electrocatalyst with good 2‐electron ORR performance over a wide pH range. The excellent performance of CNHs is due to good electron transfer and appropriate porosity.^[^ ^15^ ^]^ Lu et al. improved the 2‐electron ORR selectivity to ≈89% by implanting some oxygen‐containing functional groups on the surface of CNTs through a surface oxidation strategy. The result showed that C atom adjacent to oxygen‐containing functional group –COOH and C–O–C was the active site.^[^ ^14a^ ^]^ Kim et al. investigated the electrocatalytic H_2_O_2_ formation performance of nitrogen‐doped reduced graphene oxide (N‐rGO) materials and found that they could selectively produce H_2_O_2_ in an alkaline system at 0.70–0.10 V versus RHE.^[^ ^14g^ ^]^ Although these pioneers have made great progress in the design and identification of the active sites of heteroatom doped carbon catalysts, the catalytic performance of H_2_O_2_ production has not been satisfactory in practical applications. In addition, the performance of 2‐electron ORR catalysts can be further improved. Therefore, it is urgent to design 2‐electron ORR electrocatalyst with ultrahigh selectivity over large voltage ranges.

Herein, we successfully designed a metal‐free carbon‐based catalyst with oxygen and nitrogen comodified carbon nanotubes (N,O‐CNTs) to promote the synthesis of H_2_O_2_ in the alkaline electrolyte with 95% selectivity. Density functional theory calculations show that the N, O‐codoping could optimize the adsorption strength of *OOH and *O intermediates, and increase the selectivity and activity of the 2‐electron ORR process. This work revealed a new possibility to optimize metal‐free electrocatalysts by codoping.

## 2 Results and Discussion

### 2.1 Material Synthesis and Characterization

**Figure 1:**
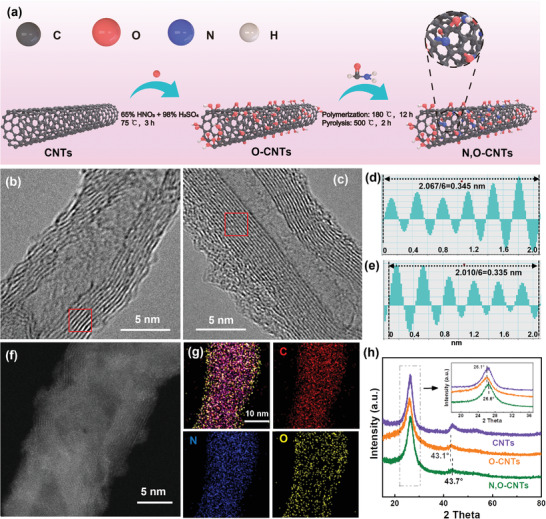
a) Schematic diagram of the synthesis route for N, O‐CNTs. HRTEM images of b) O‐CNTs and c) N,O‐CNTs. d,e) The corresponding intensity profiles of the layer spacing in (b) and (c), respectively. f) AC HAADF‐STEM image and g) corresponding EDX mapping of carbon, nitrogen, and oxygen of N,O‐CNTs. h) Powder XRD patterns of CNTs, O‐CNTs, and N, O‐CNTs.

As shown in **Figure** **1**a, nitrogen and oxygen codoped carbon nanotubes were prepared by a 3‐step synthesis strategy. Firstly, O‐CNTs were prepared by oxidizing carbon nanotubes in strong acid to introduce some oxygen‐containing functional groups such as –COOH, –COH, –C–O–C–, and –OH.^[^ ^15^ ^]^ Then the obtained O‐CNTs were coated through formamide condensation by solvothermal method. Thirdly, the N, O‐containing CNTs were pyrolyzed in the Ar atmosphere to further condense by defunctionalization to get the final product. In the second step, formamide was dehydrated and loaded as N‐rich carbon (34.59 at%, Figure S1, Supporting Information) on O‐CNTs.^[^ ^16^ ^]^ Some nitrogen atoms could be embedded into the tubular structure to form an O–C–N structure during consequent pyrolysis. The mild pyrolysis temperature (500 °C) ensured the high oxygen and nitrogen content of N, O‐CNTs.^[^ ^17^ ^]^ The oxygen and nitrogen content of the final product could be controlled by adjusting the pyrolysis temperature. In order to optimize the experimental conditions, we adopted three pyrolysis temperatures of 500, 700, and 900 °C, and the codoped carbon materials prepared were named as N, O‐CNTs, N, O‐CNTs‐700, and N, O‐CNTs‐900, respectively.

The transmission electron microscope (TEM) and high‐resolution (HR) TEM images indicated the morphology of oxidized carbon nanotubes (O‐CNTs) is essentially the same as its percussive pristine CNTs, which remains tubular structures with a diameter of 6–18 nm and a wall thickness of 2–4 nm (Figure 1b; Figures S2 and S3, Supporting Information). The HRTEM images indicated the N, O‐CNTs remain 6–18 nm in diameter after coating and pyrolysis (Figure 1c; Figure S4, Supporting Information). However, some discontinuous amorphous carbonaceous layer appears on its surface with thickness less than 3 nm (Figure 1c). The energy dispersive spectroscope (EDS) element mapping showed that N and O are uniformly distributed on N, O‐CNTs (Figure 1f,g). From the HRTEM image, it was found that the layer spacing of O‐CNTs after oxidization is ≈0.345 nm (Figure 1d), larger than the layer distance (≈0.340 nm) of pristine CNTs,^[^ ^18^ ^]^ which implied that the oxygen‐containing groups could be attached to the CNTs during oxidation, enlarge the layer spacing.^[^ ^19^ ^]^ However, when pyrolysis is applied to strengthen the N doping, the layer spacing of N, O‐CNTs decreases to ≈0.335 nm, which is smaller than the layer distance of O‐CNTs (Figure 1e). This result should be generated by the removal of part of oxygen functional groups from O‐CNTs and further condensation during the pyrolysis.

The X‐ray diffraction (XRD) spectra confirmed the shrinkage of layer spacing by showing the shift of two characteristic (002) and (100) peaks of graphite: they moved from 26.1° and 43.1° of O‐CNTs to 26.6° and 43.7° of N, O‐CNTs (Figure 1h).^[^ ^14^, ^20^ ^]^ The shift of XRD peak of N, O‐CNTs to larger angle reflects the decrease of interfacial distance, which is in good agreement with the HRTEM results.^[^ ^19^, ^21^ ^]^ For those samples after higher temperature pyrolysis, N, O‐CNTs‐700 and N, O‐CNTs‐900, they also have similar tubular structures, and their EDS mappings indicated that the content of O and N decreased with the increase of pyrolysis temperature (Figures S6 and S7, Supporting Information). Furthermore, the layer spacing presents an increasing phenomenon from ≈0.335 to ≈0.340 nm (Figure S8, Supporting Information).

**Figure 2:**
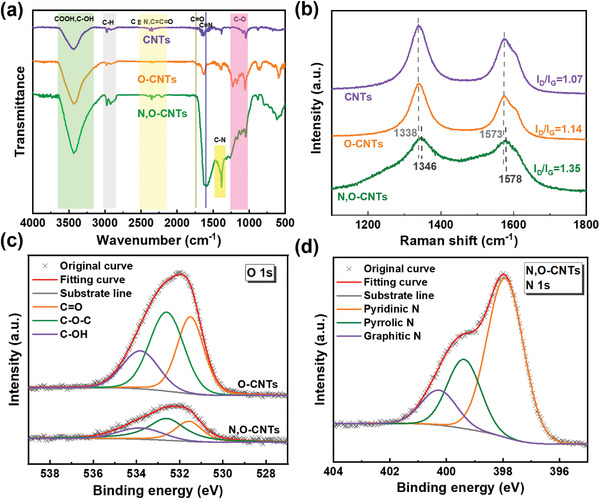
a) Background‐corrected FTIR spectra and b) Raman spectra of CNTs, O‐CNTs, and N, O‐CNTs. c) Deconvoluted oxygen 1s XPS spectra of O‐CNTs and N, O‐CNTs. d) Deconvoluted nitrogen 1s XPS spectra of N, O‐CNTs

The chemical properties of the samples were further investigated by Fourier‐transform infrared spectroscopy (FTIR), X‐ray photoelectron spectroscopy (XPS), and Raman spectroscopy. As shown in **Figure** **2**a, the content of various oxygen‐containing functional groups, such as C=O (1660–1760 cm^–1^), C–O (1014–1250 cm^–1^), and C–OH (2900–3600 cm^–1^),^[^ ^22^ ^]^ of the O‐CNTs increased. Meanwhile, the XPS spectra showed that a new peak related to O 1s appeared in both O‐CNTs and N, O‐CNTs (Figure 2c; Figures S9 and S10, Supporting Information), which could be deconvolved into the following bands: C=O (531.5 eV), C–O–C (532.6 eV), and C–OH (533.85 eV).^[^ ^17^ ^]^ Moreover, C–O (286.4 eV) and –O–C=O (288.8 eV) appeared in the deconvolution band of C 1s (Figure S10b, Supporting Information).^[^ ^23^ ^]^ These results demonstrated that O‐CNTs and N,O‐CNTs contained multiple oxygen functional groups. In addition, N, O‐CNTs had higher C–O–C content than O‐CNTs (Figure S11 and Table S1, Supporting Information). FTIR showed that C–N (1348–1462 cm^–1^) and C=N (1500–1600 cm^–1^) vibration peaks appeared in N, O‐CNTs.^[^ ^16^ ^]^ Moreover, the deconvolution of the newly emerged N 1s XPS spectrum (Figure 2d) shows three peaks: pyridine N (397.95 eV), pyrrole N (399.40 eV), and graphite N (400.30 eV).^[^ ^24^ ^]^ All the above results demonstrated the successful introduction of nitrogen and oxygen in the N,O‐CNTs.

When the pyrolysis temperature increased from 500 to 900 °C, the proportion of nitrogen element decreased from 21.59% to 2.62%, and the proportion of oxygen element decreased from 4.39% to 2.49% (Figures S12–S14; Table S2, Supporting Information). The results well agreed with the corresponding EDS mapping images (Figures S5 and S6, Supporting Information). Raman spectra of the three samples (Figure 2b) showed that the I_D_/I_G_ increased after oxygen (O‐CNTs) doping. It further increased to 1.35 after both N, O‐codoping (N,O‐CNTs), evidencing the degree of defects increased in turn.^[^ ^25^ ^]^ The Raman peak of N,O‐CNTs widened due to the increased disorder after the condensation with formamide derivated –C=N– structure materials.^[^ ^26^ ^]^ The position of D‐band shifted from 1338 cm^−1^ for pristine CNTs to 1346 cm^−1^ for N,O‐CNTs, while the position of G‐band shifted from 1573 cm^−1^ for pristine CNTs to 1578 cm^−1^ for N,O‐CNTs. The blue shift of Raman spectra was attributed to the lattice shrinkage caused by replacing carbon atoms with smaller nitrogen atoms.^[^ ^10^, ^27^ ^]^

### 2.2 Electrocatalytic ORR Performances

**Figure 3:**
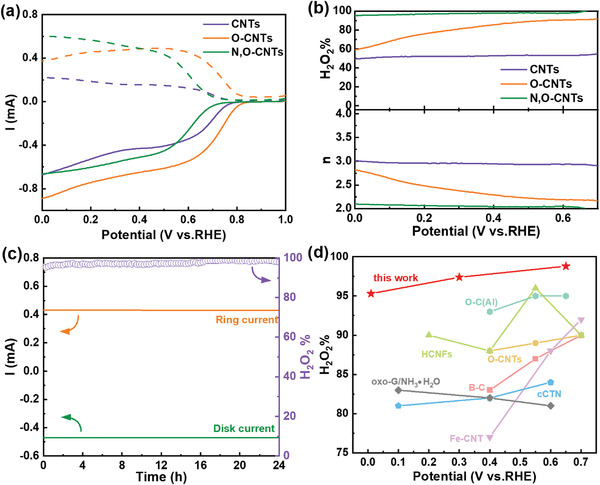
a) Illustration showing the process of O_2_ reduction to H_2_O_2_. Linear sweep voltammetry (LSV) curves of CNTs, O‐CNTs, and N, O‐CNTs (solid lines) together with the corresponding H_2_O_2_ currents on the ring electrode (dashed lines) recorded at 1600 rpm in 0.1 m KOH. b) Calculated H_2_O_2_ selectivity and electron transfer number during the potential sweep. c) Stability measurement of N, O‐CNTs at a fixed disk potential of ≈0.4 V. d) Comparison of the reactivity and selectivity for H_2_O_2_ electrosynthesis on N, O‐CNTs and other reported electrocatalysts.

The 2‐electron ORR performance was tested in a 0.1 m KOH solution saturated with oxygen at room temperature using a three‐electrode system. The collection efficiency was 0.335, as calibrated by the redox reaction of [Fe(CN)_6_]^4−^/[Fe(CN)_6_]^3–^ (Figure S15, Supporting Information).^[^ ^9c^ ^]^ **Figure** **3**a showed the linear sweep voltammetry (LSV) curves of three electrocatalysts in an alkaline solution at the oxygen saturation condition. It could be seen that N, O‐CNTs have the highest ring current within a wide voltage range (0–0.4 V vs RHE), indicating that they have higher 2‐electron ORR activity than their counterparts. Figure 3b displayed the calculated H_2_O_2_ selectivity and electron transfer number (*n*) as a function of the applied potential. The selectivity of O‐CNTs was ≈80% in 0.3–0.65 V versus RHE, much higher than that of pristine CNTs (≈50% in 0–0.65 V vs RHE), and the electron transfer number n decreased from 3 to ≈2.5. The selectivity of N, O‐CNTs was the highest in the wide voltage range 0–0.65 V versus RHE, exceeding 95%, with electron transfer number of 2.05, superior over O‐CNTs and most of the previously reported catalyst (Figure 3d; Table S3, Supporting Information). For comparison, we studied the performance of N‐CNTs in samples doped only with N. The selectivity of N‐CNTs decreased from >95% to ≈75% (0–0.65 V vs RHE), and the electron transfer number increased from 2.05 to ≈2.5 (Figure S16, Supporting Information). The above results demonstrated that the simultaneous introduction of O and N could promote the 2‐electron ORR.

Furthermore, a catalyst with excellent H_2_O_2_ selectivity should also have low electrocatalytic ability for further reduction of H_2_O_2_ (H_2_O_2_ + 2H^+^ + 2e^–^ → 2H_2_O).^[^ ^28^ ^]^ To verify the superior performance of N, O‐CNTs, we further measured the electrochemical reduction of H_2_O_2_ in a KOH electrolyte containing 1 × 10^−3^ m H_2_O_2_. As shown in Figure S17 (Supporting Information), when the N, O‐CNTs were used as catalysts, H_2_O_2_ electrochemical reduction current was less than 0.04 mA in the entire voltage range. The durability was further evaluated by chronoamperometric responses. When N, O‐CNTs were continuously used to produce the H_2_O_2_ for 24 h, the ring current and disk current remained stable, and the high selectivity of ≈95% could be maintained throughout the whole process (Figure 3c). Thereby, through codoping of oxygen and nitrogen, N, O‐CNTs showed the highest selectivity, widest work voltage range, and longest durability.

However, it should be noted that the high performance of N, O‐CNTs can only be achieved on 500 °C calcined sample. Higher pyrolysis temperature could lead to the decline of its 2‐electron ORR performance, accompanied by the decrease of O and N contents. After 900 °C pyrolysis, the selectivity of H_2_O_2_ generated by electrochemical ORR is only about 40% (Figure S18, Supporting Information).

### 2.3 DFT Calculation Revealed the Catalytic Performance

N,O‐CNTs have the highest specific electrochemical activity surface area (Figure S20, Supporting Information), which is one possible reason for better performance, but insufficient to explain the high intrinsic reactivity and selectivity toward the 2‐electron ORR with the oxygen and nitrogen codoping. We thus conducted density functional theory (DFT) calculations to identify the roles of oxygen and nitrogen in tailoring the catalytic activity of CNTs.

**Figure 4:**
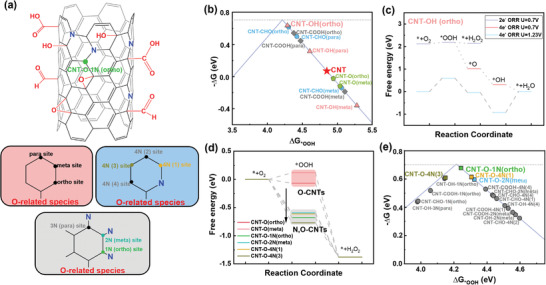
a) DFT calculation models with potential active sites for electrochemical production of H_2_O_2_. Carbon atoms represented by solid dots are active sites for study. The green solid point is the optimal active site for H_2_O_2_ production of N, O‐CNTs (The detailed model diagram was shown in Figures S21–S25 (Supporting Information). b) The ORR simulated activity volcano plot of O‐CNTs. The theoretical equilibrium potential is shown as a gray dashed line. c) Free energy diagram of 2‐electron/4‐electron ORR on the CNT‐OH (ortho) site. d) Calculated the change of free energy of 2‐electron/4‐electron ORR after doped N on the single‐O‐doping nanotube sites (O‐CNTs). e) The ORR simulated activity volcano plot of partial sites of N, O‐CNTs.

Firstly, we demonstrate the O‐doping can regulate the adsorption ability of vicinal C atom for ORR intermediates (*OOH, *OH, and *O). The possible catalytic sites on the CNTs considering COOH*, CHO*, *OH, and *O were considered, as shown in **Figure** **4**a and Figures S21–S25 in the Supporting Information. To verify how oxygen‐containing functional groups bind to CNTs, we calculated and compared the adsorption energy of O‐adsorbed and O‐embedded CNTs as displayed in Figure S26 in the Supporting Information. We found that the O‐adsorbed CNTs is more stable than the O‐embedded CNTs. Especially with increasing O content, the *O adsorption is favorable thermodynamically. Therefore, the oxygenated species prefers to adsorb on the CNTs rather than be embedded into CNTs rather than be embedded into CNTs. We further investigated 2‐/4‐electron ORR on these models (Figures S27–S31, Supporting Information) and constructed a volcano plot shown in Figure 4b of the 2‐electron ORR activity as a function of the *OOH adsorption energy (ΔG_*OOH_) and calculated the 2‐electron and 4‐electron ORR activities of all possible sites (O‐CNTs) in Figure 4a and Figure S21 (Supporting Information).^[^ ^17^ ^]^ We found that the CNTs have an inferior 2‐electron ORR activity due to its relatively weak ΔG_*OOH_ of 4.94 eV (red star in Figure 4b). Among all sites on O‐CNTs, the ortho‐/para‐ carbon sites of COOH*, CHO*, and *OH endow high catalytic activity, whereas all carbon sites close to *O and meta‐ carbon sites adjacent to other O‐related species exhibit low ΔG_*OOH_ compared to CNTs. Clearly, introducing O‐containing species can effectively regulate the reactivity of carbon sites close to the carbon atoms bound to O‐related species and lead to the generation of highly catalytic sites, which was in consistence with our experimental results that oxidized CNTs showed higher ORR activity. Furthermore, compared with 2‐electron ORR and 4‐electron ORR process under the standard potential of 0.7 and 1.23 V, respectively, the CNT‐OH (ortho) site possessed a low 2‐electron ORR overpotential (0.06 V) and relatively high 4‐electron ORR overpotential (0.60 V), indicative of kinetically high selectivity for 2‐electron ORR process (Figure 4b). However, under the working voltage of 0.7 V, the second proton/electron step thermodynamically prefers the conversion of *OOH into *O, resulting in the accumulating of *O on active sites and lower the density of catalytic sites. Besides, the in situ formed *O site will likely behave as CNT‐O, which was speculated to reduce the 2‐electron ORR activity. Therefore, O‐doping can selectively facilitate the catalytic activity of the vicinal C sites but cannot inhibit the site poisoning phenomenon caused by *O adsorption during ORR process.

Then, based on the comprehension of O‐doping effects, we investigated the improvement effect of N‐doping on CNTs. Considering the higher thermodynamical stability and stronger regulation ability of graphite N compared to that of pyrrole N and pyridine N (Figure S32, Supporting Information), we select graphite N to integrate into O‐doping CNT displayed in Figure 4a and Figures S22–S25 in the Supporting Information. Then, the free energy changes of 2‐/4‐electron ORR process were conducted on N,O‐codoping CNT. Surprisingly, the introduction of N significantly increases the *OOH adsorption of CNT‐O and thus generates highly catalytic sites: CNT‐O‐1N (ortho), CNT‐O‐2N (meta), CNT‐O‐4N (1), and CNT‐O‐4N (3), which corresponding overpotentials were 0.01, 0.09, 0.08, and 0.08 V, respectively (Figure 4d). Such newly emerged catalytic sites possess the peak of 2‐electron volcano plot exhibited in Figure 4e and Figure S33 (Supporting Information), providing higher catalytic activity with lower overpotential. Furthermore, the inclusion of N also strengthens the adsorption of oxygenated species on CNTs and tailor the charge density of adjacent carbon sites (Figure S34, Supporting Information). This leads to the formation of CNT‐O sites adjacent to N sites during ORR process and tailor the electronic structure of carbon sites around N atoms. These newly emergent active sites provided enhancing *OOH adsorption for improving 2‐electon ORR and weaker *O adsorption for lowering the site poisoning effect (Figure S35a, Supporting Information), which can provide a large number of highly active sites. Therefore, the N, O codoping not only effectively alleviates the degradation of catalytic activity caused by epoxide group poisoning of O‐doping CNT, but also generates highly catalytic sites combining epoxide group and graphite N. The cooperation of O‐ and N‐doping is responsible for the excellent 2‐electron ORR catalytic performance of N, O‐CNTs.

### 2.4 The ORR Performance Evaluated in H‐Cell

**Figure 5:**
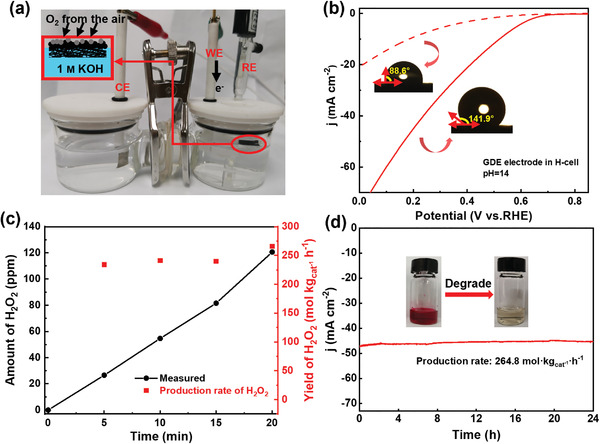
a) The electrochemical device used for the electrochemical synthesis of H_2_O_2_. Detailed: the image of Janus electrode floating over the electrolyte with the aerophilic (AI) side upward and aerophobic (AO) side downward. The electrolyte is 1 m KOH. b) LSV of N, O‐CNTs catalyst through Janus electrode in 1 m KOH via an H‐cell electrolyzer after spraying PTFE (solid line) and un spraying PTFE (dashed line) and the corresponding contact angle (inset). c) The amount of generated H_2_O_2_ and production rate of H_2_O_2_ along with reaction time at a cell output voltage of 0.15 V in 1 m KOH. d) Stability test in 1 m KOH. The Insert is the degradation process demonstration of fuchsin basic with the generated H_2_O_2_.

Besides activity, the durability or stability of catalysts is also crucial for its application. We check the stability of N,O‐CNTs in three‐electrode electrolysis in H‐cell. The catalyst was loaded with 2 mg cm^–2^, a layer of polytetrafluoroethylene (PTFE) solution was deposited on the same side (Figures S36 and S37, Supporting Information) to make the catalyst side hydrophobic to construct Janus electrode which can work both as a gas diffusion electrode (GDE) and the working electrode.^[^ ^29^ ^]^ It was found that the droplet contact angle increased from 88.6° to 137° after PTFE modification (**Figure** **5**b, illustration). Then, the Janus electrode with asymmetric wettability was used to conduct electrolysis in H‐cell. A schematic diagram of the device was shown in Figure 5a and Figure S38 (Supporting Information). The device only needs air to complete the generation of H_2_O_2_. When PTFE was not deposited for electrolysis, the polarization current was 10 mA cm^–2^ at 0.2 V versus RHE. When PTFE is sprayed, the polarization current could reach 43 mA cm^–2^, more than four times the original (Figure 5b). Meanwhile, the Janus electrode could continuously increase the current within a specific ORR voltage range (Figure S39, Supporting Information), revealing the impact of PTFE modification.

One challenging thing for in situ H_2_O_2_ production is that H_2_O_2_ generated on cathode might be further reduced to H_2_O due to its slow diffusion,^[^ ^30^ ^]^ resulting in decreased H_2_O_2_ yield. However, N, O‐CNTs showed very inert performance for H_2_O_2_ reduction. Under an electrolytic voltage of 0.15 V versus RHE, the device can achieve continuous growth within 20 min, demonstrating that the production rate of H_2_O_2_ is much higher than the decomposition rate of H_2_O_2_. Moreover, the H_2_O_2_ productivity remained stable in this progress and was calculated to be 265.8 mol kg_cat_^–1^ h^–1^ at 20 min (Figure 5c; Figures S40 and S41, Supporting Information). Chronoamperometric measurement evaluated the stability of the catalyst. Continuous production with a yield of 264.8 mol kg_cat_^–1^ h^–1^ can be achieved within 24 h and presented a very stable state (Figure 5d; Figure S42, Supporting Information). The electrolytic solution was mixed with 1 × 10^−3^ m fuchsin basic solution in a 3:1 volume ratio and the magenta solution got bleached immediately (inset), demonstrating its potential application prospects.

## 3 Conclusion

In summary, we reported a nitrogen and oxygen comodified carbon nanotubes (N, O‐CNTs) electrocatalyst, which exhibited excellent H_2_O_2_ selectivity for electrocatalytic 2‐electron oxygen reduction in the wide voltage range from 0 to 0.65 V versus RHE. More importantly, it has low electrocatalytic ability for further reduction of H_2_O_2_ to water. DFT calculations indicated that the O‐doping could effectively increase the 2‐electron ORR activity, while N‐related species further decrease the deactivation due to oxygen accumulation and thus provides stable and highly catalytic sites. The O‐ and N‐species altogether optimized *OOH adsorption of CNTs and leaded to high selectivity. Its stability was confirmed by yielding H_2_O_2_ at a rate of 264.8 mol kg_cat_^–1^ h^–1^ for 24 h. Taking advantage of the excellent performance of N, O‐CNTs, we assembled H‐cells of the Janus electrode for the working electrode, which did not require additional supplemental oxygen. The heteroatomic cocatalysis strategy should pave a new way for optimizing carbon‐based catalysts for electrochemical catalysis.

## Conflict of Interest

The authors declare no conflict of interest.

## Supporting information

Supporting Information

## Data Availability

The data that support the findings of this study are available from the corresponding author upon reasonable request.
