# Supplementary material for: Synergistic Effects in N,O‐Comodified Carbon Nanotubes Boost Highly Selective Electrochemical Oxygen Reduction to H2O2
Source: Adv Sci (Weinh). 2022 Jul 28;9(27):2201421. doi: 10.1002/advs.202201421 (PMC9507382; doi:10.1002/advs.202201421)
Supplement: Supplementary file 1 — Supporting Information [file ADVS-9-2201421-s001.pdf]

ADVANCED SCIENCE
Open Access

Supporting Information

for *Adv. Sci.*, DOI 10.1002/advs.202201421

Synergistic Effects in N,O-Comodified Carbon Nanotubes Boost Highly Selective Electrochemical Oxygen Reduction to H_2O_2

Shuhui Xu, Ruihu Lu, Kai Sun, Jialun Tang, Yaping Cen, Liang Luo, Ziyun Wang, Shubo Tian* and Xiaoming Sun**

Supporting Information

1. Experimental section

1.1. Synthesis of O-CNTs：

A traditional process. 250 mg CNTs were mixed with 45 mL oxidizing acid (98 wt.% H_2SO_4: 65 wt.% HNO_3 = 3:1, volume ratio), stirred at 75 ºC for 3 h, cooled, centrifuged, and freeze-dried.

1.2. Synthesis of N,O-CNTs, N,O-CNTs-700, N,O-CNTs-900 and N-CNTs：

O-CNTs (100 mg) were uniformly dispersed in formamide solution (70 mL) by ultrasound. The mixture was then transferred to a 100 mL Teflon-lined autoclave, sealed, and reacted at 180 ºC for 12 h. After solvothermal treatment, N,O-CNTs-0 was obtained by washing and drying with water and ethanol. The dried sample N,O-CNTs-0 were ground in an agate mortar and placed in a quartz crucible. The quartz crucible was then transferred to a tubular furnace under Ar for heating at a heating rate of 5 ℃ min^{-1} and annealed for 2 h. Different pyrolysis temperatures of 500 ºC, 700 ºC, and 900 ºC correspond to samples N,O-CNTs, N,O-CNTs-700 and N,O-CNTs-900, respectively. In the preparation of N-CNTs, precursor CNTs were directly solvothermal reacted with formamide at 180 ºC without the oxidation process in Step 1.1, and then pyrolyzed in a tubular furnace at 500 ºC in Ar atmosphere. The pyrolysis parameters were the same as above. After natural cooling to room temperature, the black product generated after grinding was used to prepare catalyst ink.

1.3. Characterization

Morphologies and structure of as-obtained samples were conveyed using field emission scanning electron microscopy (SEM, Zeiss SUPRA 55, operated at 20 kV) and transmission electron microscopy (TEM, FEI Tecnai G2 20 STwin, operated at 200 kV). High-resolution transmission electron microscopy (HRTEM, operated at 200 kV) images and element mapping images were recorded using a JEOL 2100 high-resolution transmission electron microscope. Aberration-corrected high-angle annular darkfield scanning transmission electron microscope (ACHAADF-STEM) images and element mapping images were recorded on a JEOL JEM-ARM200F TEM/STEM with a spherical aberration corrector (operated at 200 kV). Fourier transform infrared spectroscopy (FTIR) spectra were analyzed on a Bruker Tensor II infrared spectrometer with KBr pellets. Raman spectra were recorded on a LabRAM Aramis Raman spectrometer (HORIBA Jobin Yvon). Powder X-ray diffraction (XRD) patterns were

recorded on an X-ray powder diffractometer (MeasSrv F9XDZ42) with Cu Kα (λ = 0.154 nm) radiation at a scan rate of 10 ºC min-1 in the 2θ range from 5 to 80°. X-ray photoelectron spectroscopy (XPS) was recorded on a Thermo Electron ESCALAB250 XPS Spectrometer (X-ray Source: Al). The C1s line at 284.8 eV was utilized to reference the binding energies in the acquired spectra. The concentration of Ti^{4+} was measured by UV-vis spectrometer (Shimadzu 2600) at 408 nm.

1.4. Electrochemical measurements

Electrochemical data were collected using WaveDrive 10 electrochemical workstation (Pine Research Instrumentation, USA). A three-electrode system was used for electrochemical measurement. The saturated calomel electrode (SCE) was used as a reference electrode, platinum plate electrode was used as the opposite electrode, disk area was 0.247 cm^{-2}, and the working electrode was a platinum ring (ring area was 0.168 cm^{-2}). ORR occurs on the disk electrode, and H_2O_2 generated in situ is continuously oxidized on the ring electrode at 1600 rpm. All potentials measured at SCE were converted to reversible hydrogen electrode (RHE) potentials. To prepare the working electrode, a 4.1 mg sample and 20 µL Nafion solution (D520, 5%) were added into 0.98 mL isopropyl alcohol under ultrasonically mixing to get the homogeneous ink. RRDE measurement was then carried out by uniformly dropping 6 µL ink onto the disk electrode using a rotary coating device. LSV test was performed in O_2-saturated 0.1 M KOH solution with a scan rate of 5 mV s^{-1} at 1600 rpm. The applied potential on Pt ring during the LSV test was set as 1.5 V versus RHE to record the ring current.[1] Before every LSV test, successive cycle voltammetry (CV) with a rapid scan rate in the potential range of 0-1.0 V versus RHE was carried out on the Pt ring of RRDE to fresh the ring electrode. By measuring the oxidation-reduction reaction of $[Fe(CN)_6]^{4-}/[Fe(CN)_6]^{3-}$, the platinum ring capture efficiency (N) was calculated to be 0.335.

The H_2O_2 selectivity and the electron transfer number (n) were calculated based on the following equations:

$$H_2O_2\% = \frac{200 \times I_{ring}/N}{|I_{disk}| + I_{ring}/N}$$

$$n = \frac{4|I_{disk}|}{|I_{disk}| + I_{ring}/N}$$

Where I_{ring} is the ring current, I_{disk} is the disk current and N is the collection efficiency of the RRDE.

The electrochemically active surface area was measured by double layer capacitance method. CV scans were conducted at the potential window from 0.96 to 1.04 V versus RHE reference electrode with scan rates of 5, 10, 15, 20, and 25 mV s^{-1}. By plotting the (Ja − Jc)/2 at 1.0 V versus RHE against the scan rate (Ja is the anodic current density and Jc is the cathodic current density), the slope value was calculated to be the double layer capacitance (C_{dl}).

1.5. Wettability regulation of membrane electrode

The preparation process of asymmetric wettability Janus electrode is shown in Figure S16. Hydrophobic carbon paper (purchased from Toray) was cleaned with hydrochloric acid, distilled water, and then ethanol with ultrasonication and dried at 60 ºC. 2.0 mg N,O-CNTs and 10 µL Nafion solution (D520, 5%) were added into 0.99 mL isopropyl alcohol under ultrasonically mixing to get the homogeneous ink. Drop 1 mL ink evenly onto one side of the cleaned carbon paper and dry the electrode under an infrared lamp. The carbon paper was then transferred to a heating table at 70 ºC, and 1mL of 1 wt.% polytetrafluoroethylene (PTFE) solution was evenly sprayed on the side containing the catalyst and roasted at 350 ºC for 30 min in a tubular furnace

1.6. H_2O_2 concentration measurement

The electrochemical measurement of H_2O_2 production was performed using a custom H-cell. As shown in Figure S18, the Janus electrode was used as the working electrode to electrochemical catalysis produce H_2O_2 in a two-compartment cell. The counter electrode is a platinum sheet electrode, the reference electrode is a saturated calomel electrode, the electrolyte is 1.0 M KOH, and Nafion 117 membrane is a separator.

The H_2O_2 concentration was measured by a traditional titration method based on the mechanism that a colorless solution of Ti $(SO_4)_2$ would be oxidized by H_2O_2 to H_2TiO_4 with yellow color in neutral and acidic environments.

$$Ti^{4+}+H_2O_2+2H_2O \rightarrow H_2TiO_4+4H^+$$

Thus, the concentration of H_2O_2 after the reaction can be measured by UV-Vis spectroscopy. The wavelength used for the measurement of H_2TiO_4 was 408 nm. In order to build an acidic environment, 10 mM Ti $(SO_4)_2$ and 2.0 M H_2SO_4 were mixed evenly as a chromogenic agent. H_2O_2 with a known concentration of 24, 48, 96, 120 ppm was mixed with the chromogenic agent at a volume ratio of 1:1. The absorbance of its UV-Vis at 408 nm was measured. After linear fitting, the standard curve was drawn (Figure S19). During the H-cell electrolysis of N,

O-CNTs, the electrolyte at 0, 5, 10, 15, and 20 min was taken for color development, and its absorbance was measured and incorporated into the standard curve to obtain the actual H_2O_2 content.

2. Computational details

All DFT calculations were constructed and implemented in the Vienna ab initio simulation package (VASP).[2] Using the electron exchange and correlation energy was treated within the generalized gradient approximation in the Perdew−Burke−Ernzerhof functional (GGA-PBE) (GGA-PBE),[3] the calculations were done with a plane-wave basis set defined by a kinetic energy cutoff of 450 eV. The K-point sampling was obtained from the Monkhorst−Pack scheme with a (3 × 3 × 1) mesh for optimization. The geometry optimizations and energy calculations are converged when the electronic self-consistent iteration and force reach 10^{-5} eV and 0.02 eV $Å^{-1}$, respectively.

2.1. Models

The diameter of nanotube is 5.42 Å. The target nanotube supercell with a lattice constant of 15.00 × 15.00 × 9.85 $Å^3$, contains 80 carbon atoms (Labelled as CNT). And for constructing CNT-COOH, CNT-CHO, CNT-OH, and CNT-O models, the *COOH, *CHO, *OH, and *O adsorption on pristine nanotube is built. In addition, we introduce a N atom to replace a C atom of the nanotube to establish N-doping nanotube (Labelled as CNT-N). According to Figure 4(a), we built a series of combinations of N, O-doping nanotube (Labelled as CNT-O-N).

2.2. The Gibbs Free Energy Variation

The change in Gibbs free energy (△G) of each adsorbed intermediate is calculated based on the computational hydrogen electrode method developed by Nørskov et al.[4] At standard condition (T = 298.15 K, pH = 0, and U = 0 V (vs. SHE)), the free energy G is defined as the following equation:

$$\Delta G = \Delta E + \Delta E_{ZPE} - T\Delta S$$

Where $\triangle E$ is the energy change obtained from DFT calculation, $\triangle E_{ZPE}$ is the difference between the adsorbed state and gas, which was calculated by summing vibrational frequency for all models based on the equation: $E_{ZPE} = 1/2\sum h\nu_i$ (T is the temperature (298.15 K) in the above reaction system, and △S represents the difference on the entropies between the

adsorbed state and gas phase. The entropies of free molecules were obtained from NIST database (https://janaf.nist.gov/).

2.3. Adsorption energy

Including *OOH, *O and *OH, it was calculated relative to H_2O and H_2 under conditions of T = 298.15 K, pH = 0, and U = 0 V (vs. SHE) according to following equations:

$$\Delta E_{*OOH} = E_{*OOH} + 3/2\, E_{H_2} - E_{*} - 2\, E_{H_2O}$$

$$\Delta E_{*O} = E_{*O} + E_{H_2} - E_{*} - E_{H_2O}$$

$$\Delta E_{*OH} = E_{*OH} + 1/2\, E_{H_2} - E_{*} - E_{H_2O}$$

Where * represents the adsorption sites associated with nanotube. The above △Gads are defined as the reaction free energies of the following reactions.

$$* + 2H_2O \rightarrow *OOH + 3/2H_2$$

$$* + H_2O \rightarrow *O + H_2$$

$$* + H_2O \rightarrow *OH + 1/2H_2$$

3. Supplementary Figures

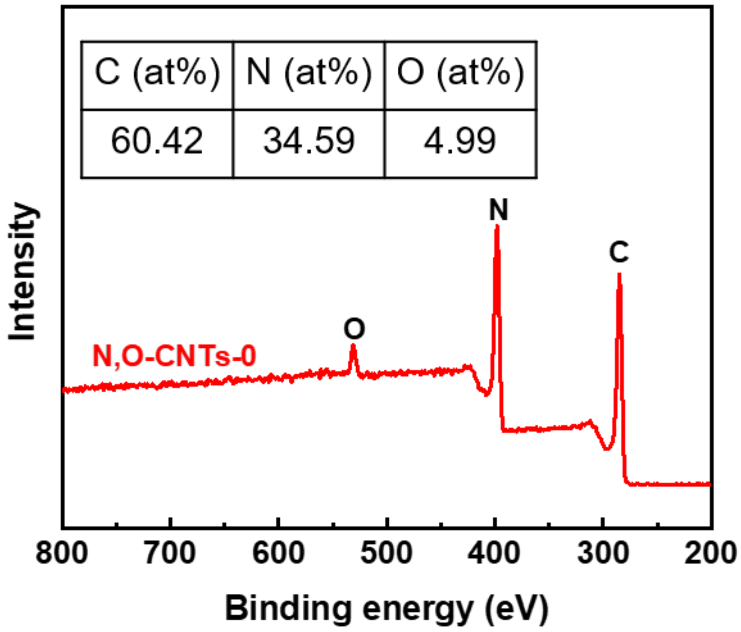

Figure S1. XPS survey spectra of N,O-CNTs-0. N,O-CNTs-0 is the material that has not been pyrolyzed.

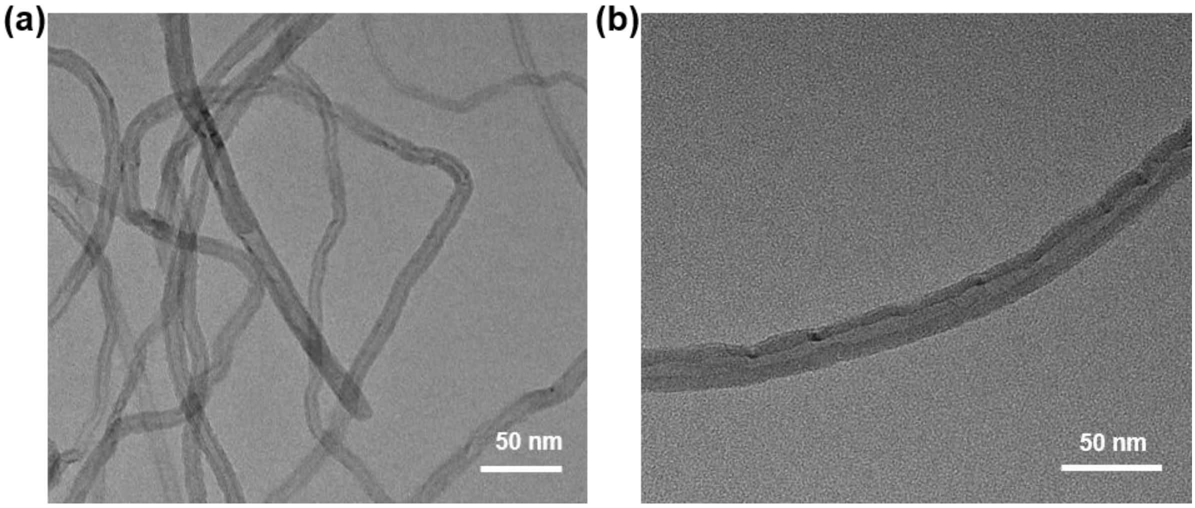

Figure S2. TEM image of CNTs.

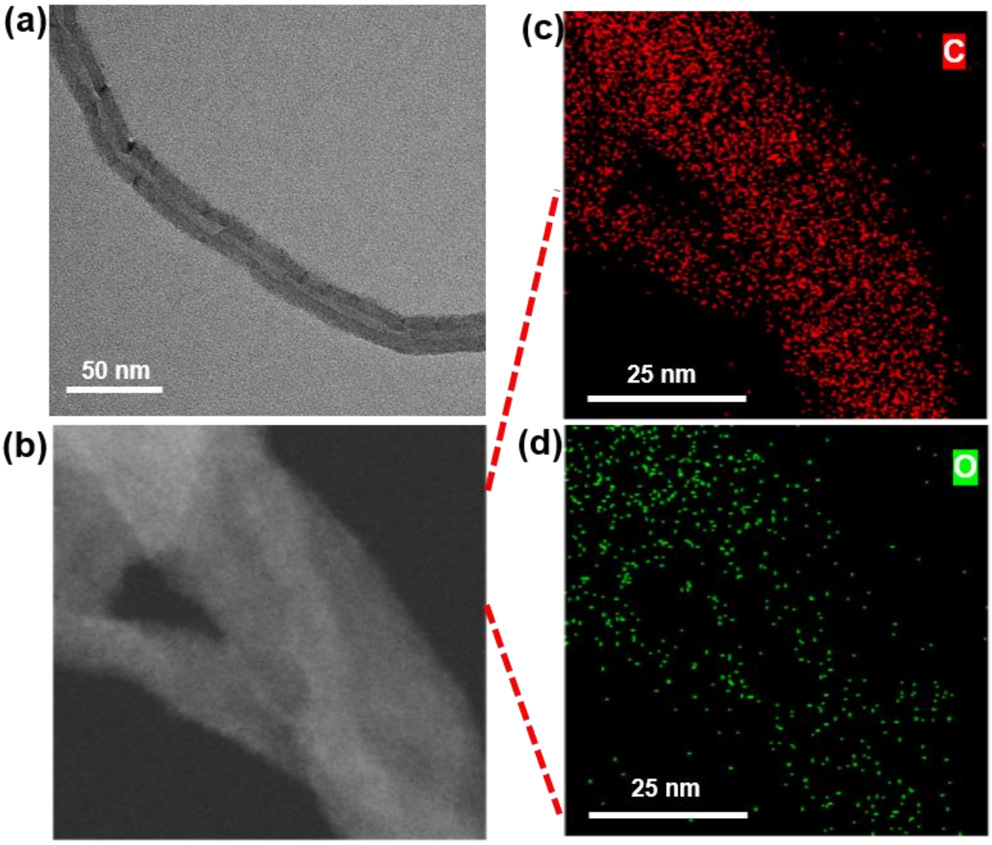

Figure S3. (a) TEM image and (b) HRTEM image and (c) (d) corresponding EDX mapping of carbon and oxygen of O-CNTs.

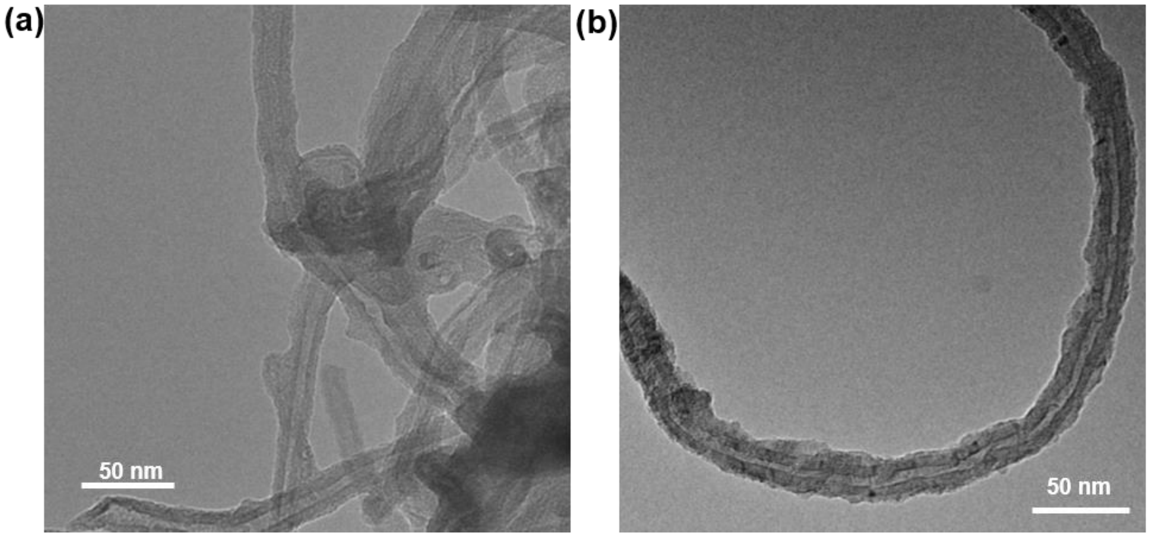

Figure S4. TEM images of N,O-CNTs.

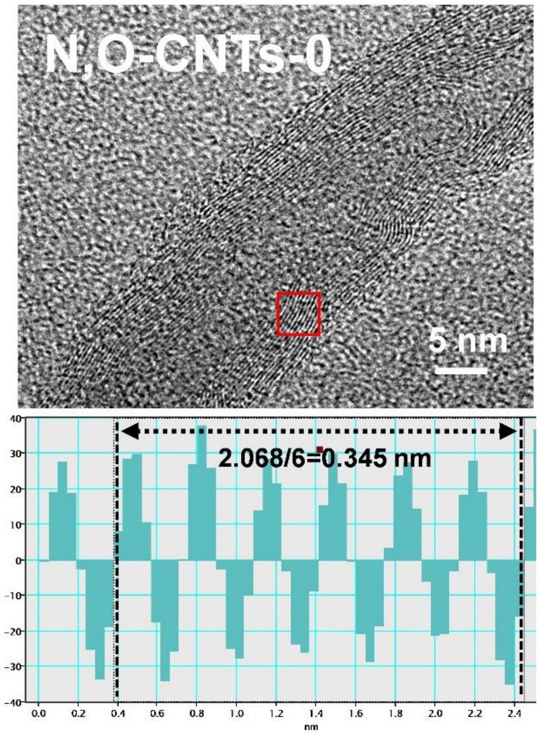

Figure S5. HRTEM images of N,O-CNTs-0 without pyrolysis and the corresponding intensity profiles of the layer spacing.

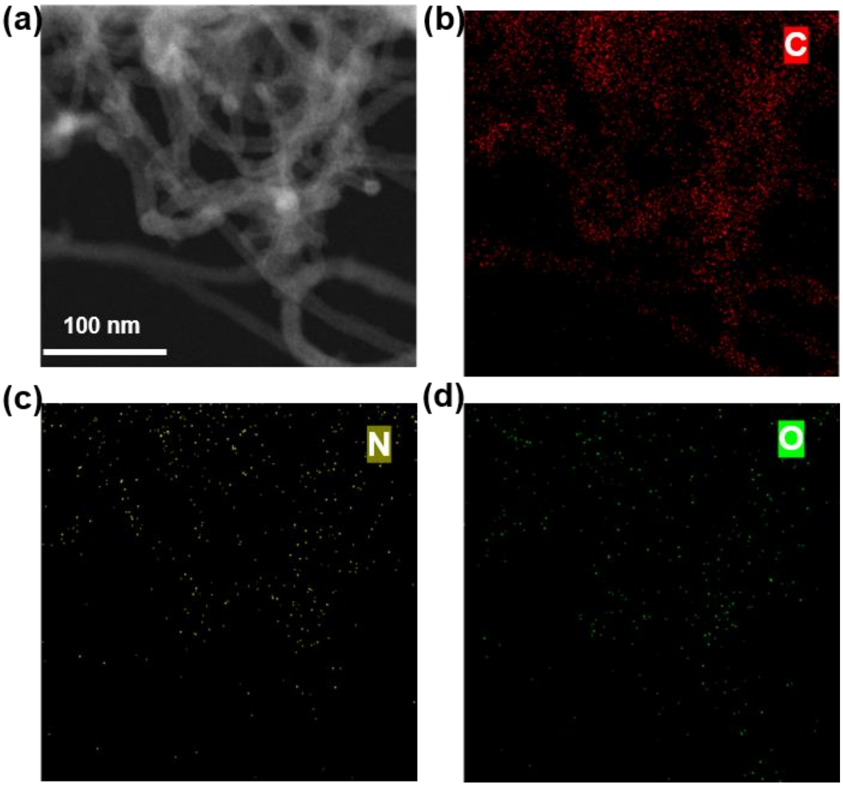

Figure S6. (a) HRTEM image and (b)(c)(d) corresponding EDX mapping of carbon, nitrogen, and oxygen of N,O-CNTs-700.

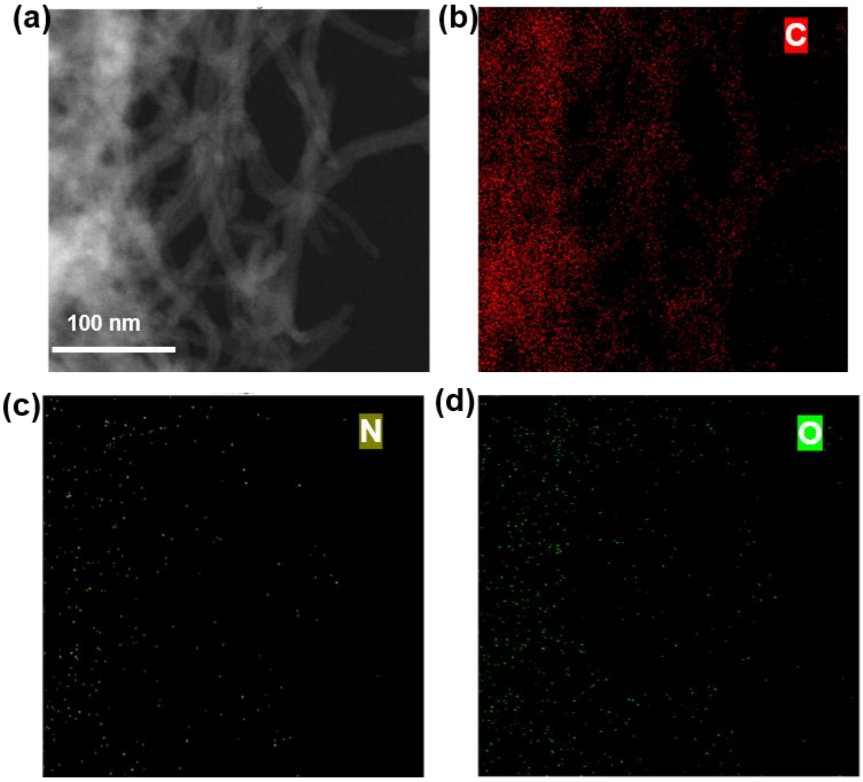

Figure S7. (a) HRTEM image and (b)(c)(d)corresponding EDX mapping of carbon, nitrogen, and oxygen of N,O-CNTs-900.

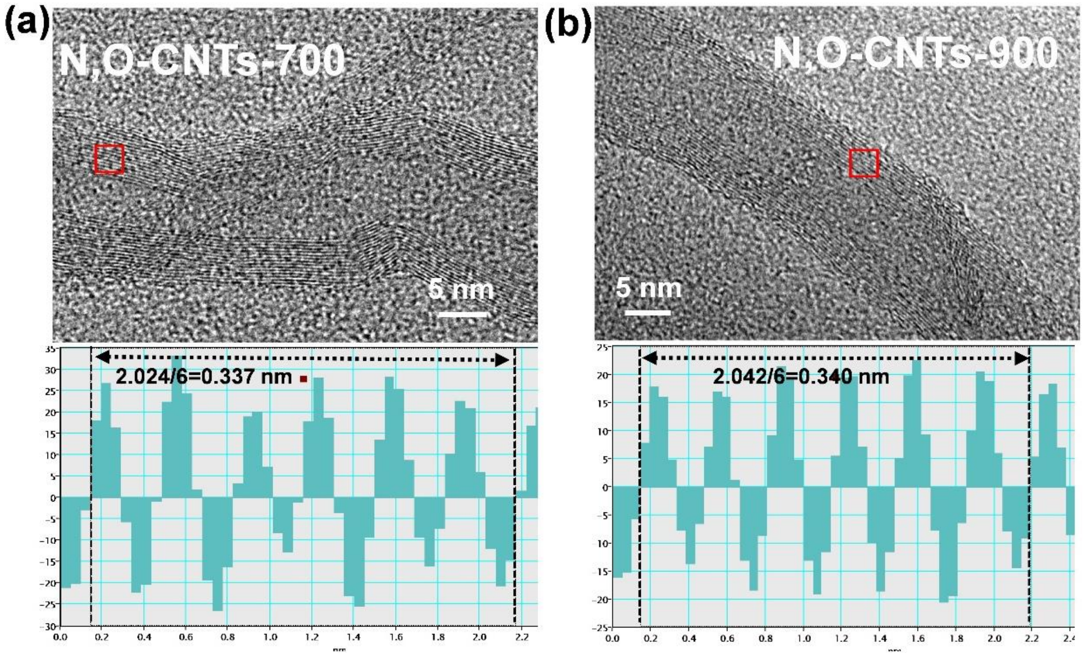

Figure S8. HRTEM images of (a) N,O-CNTs-700, (b) N,O-CNTs-900 and the corresponding intensity profiles of the layer spacing.

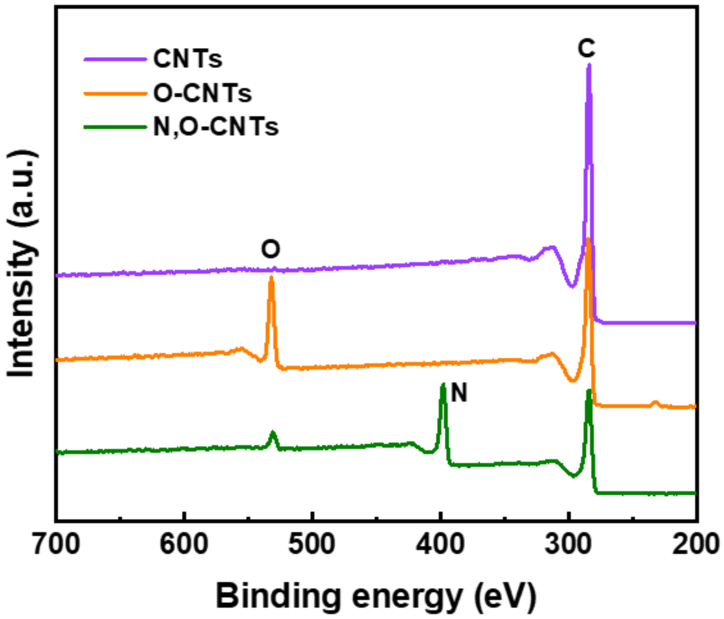

Figure S9. XPS survey spectra of CNTs, O-CNTs and N,O-CNTs. C (284.8 eV), N (399 eV) and O (532.2 eV) elements are detected.

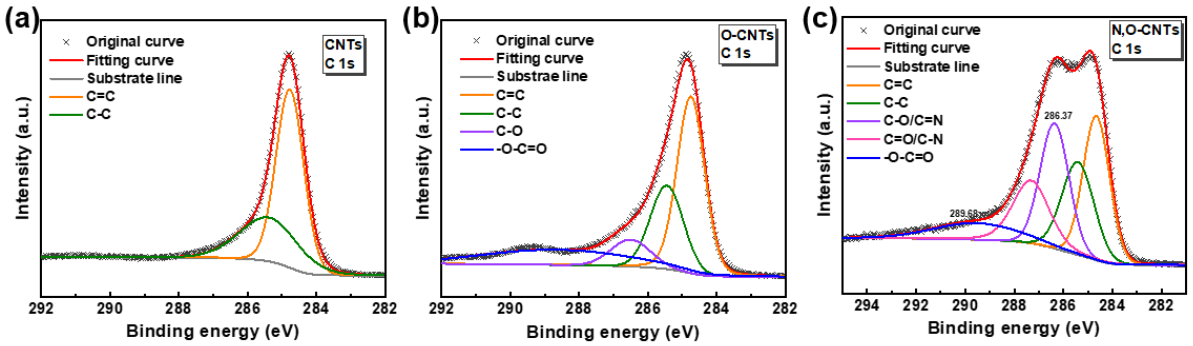

Figure S10. Deconvoluted carbon 1s XPS spectra of CNTs, O-CNTs, and N,O-CNTs.

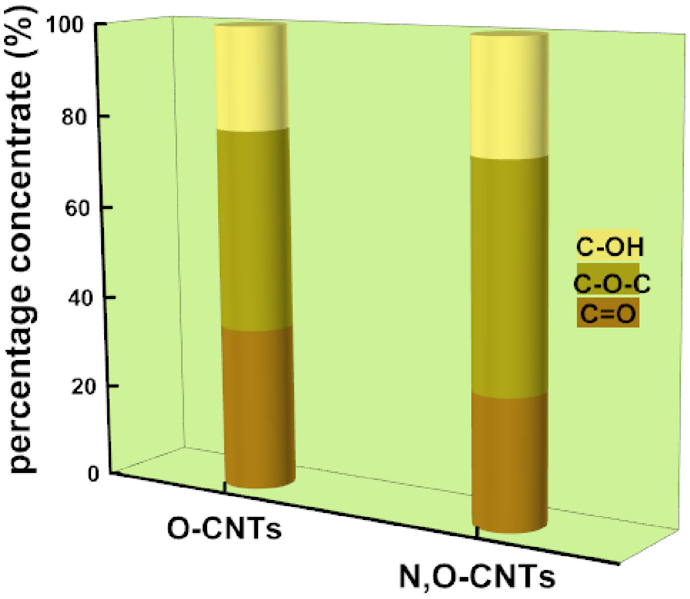

Figure S11. Bar chart of percentage concentrations of different oxygen species for O-CNTs and N,O-CNTs.

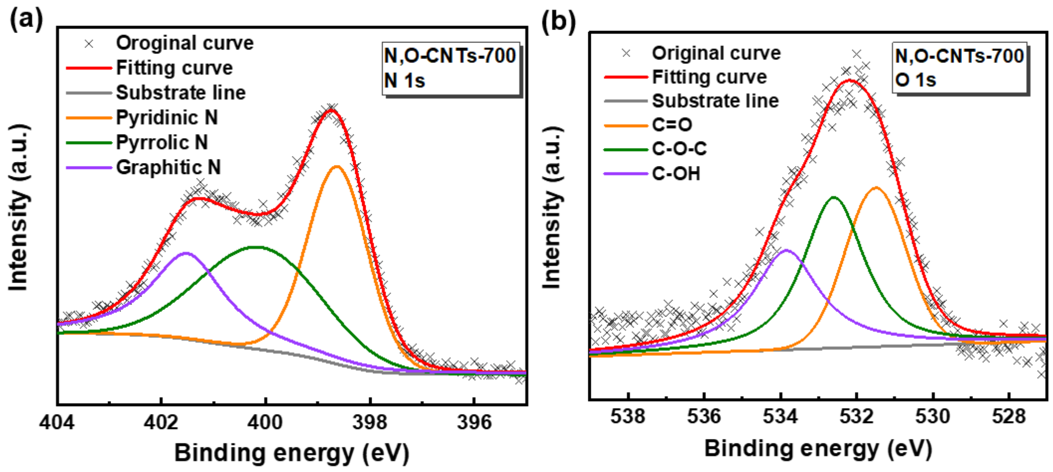

Figure S12. Deconvoluted N 1s and O 1s XPS spectra of N,O-CNTs-700.

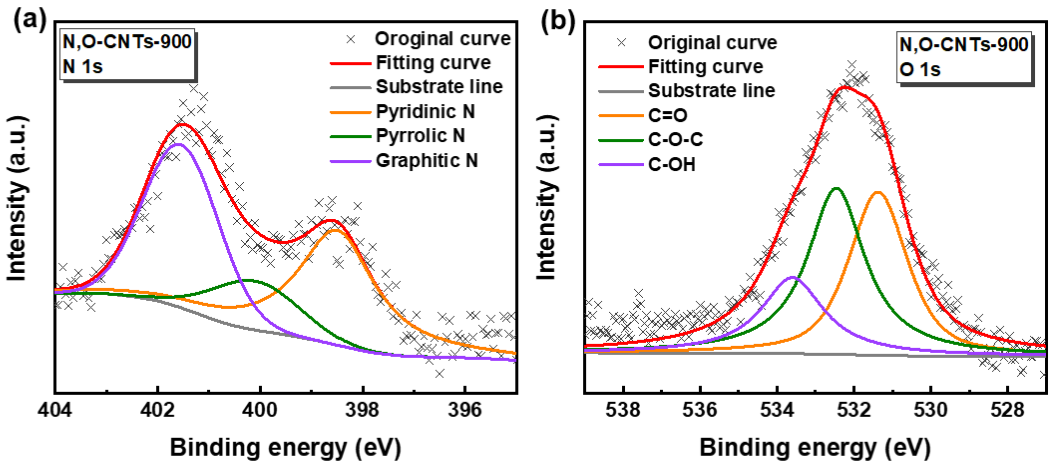

Figure S13. Deconvoluted N 1s and O 1s XPS spectra of N,O-CNTs-900.

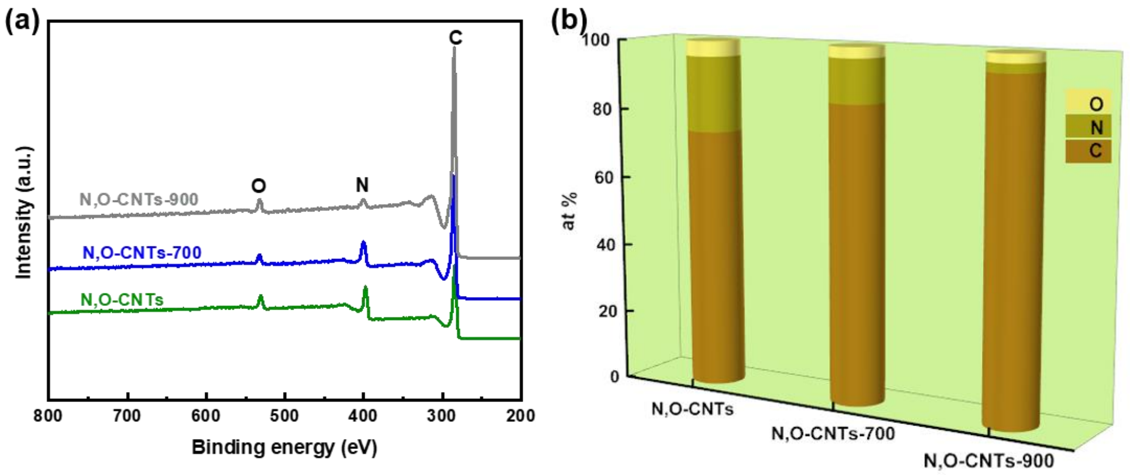

Figure S14. (a) XPS survey spectra and (b) Bar chart of atomic content of N,O-CNTs, N,O-CNTs-700, and N,O-CNTs-900. C (285 eV) , N(399 eV) and O (532.2 eV) elements are detected.

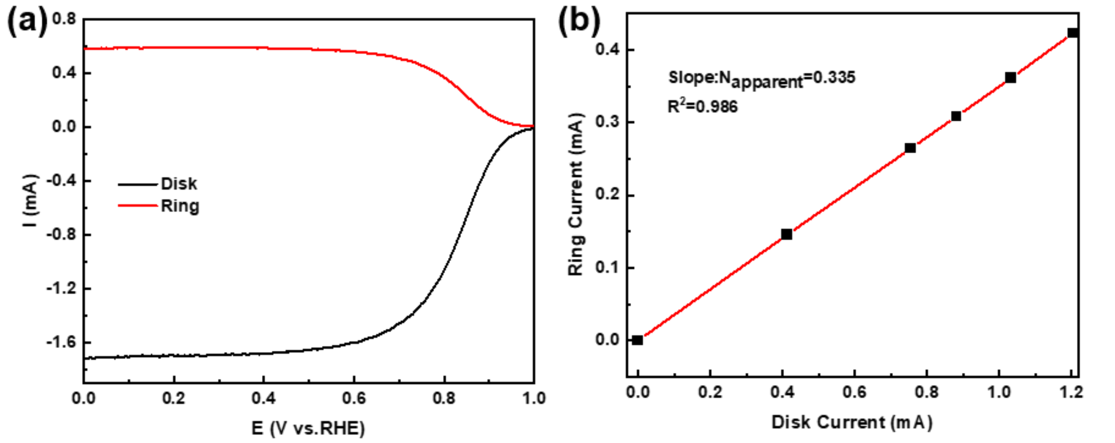

Figure S15. Linear sweep voltammetry curves were recorded on a bare glassy carbon rotation disk electrode with a Pt ring in the electrolyte containing 0.5 M KCl and 10 mM K_3Fe $(CN)_6$ at 1600 rpm. Counter electrode: C rod, reference electrode: saturated calomel electrode (SCE), Ring potential: 1.5 V vs. RHE. (b) Linear fitting of the diffusion limited current densities recorded on ring and disk electrodes at 1600 rpm. The experimental determined apparent collection efficiency (N) is 33.5%.

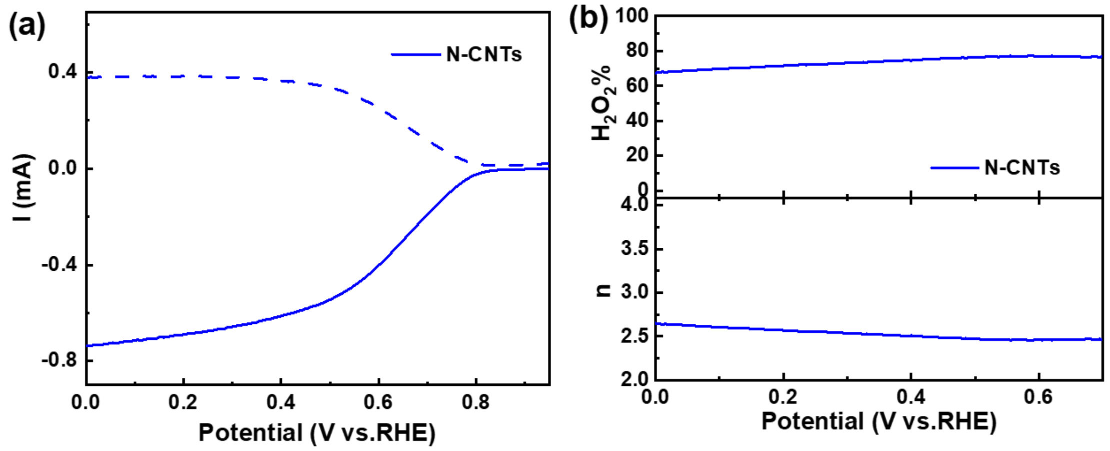

Figure S16. (a) Illustration showing the process of O_2 reduction to H_2O_2. Linear sweep voltammetry (LSV) curves of N-CNTs (solid lines) together with the corresponding H_2O_2 currents on the ring electrode (dashed lines) recorded at 1600 rpm in 0.1 M KOH. (b) Calculated H_2O_2 selectivity during the potential sweep.

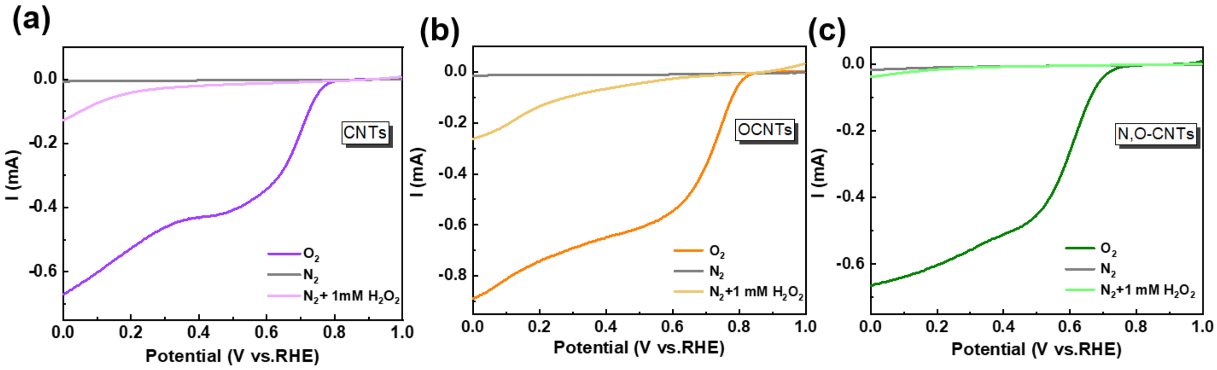

Figure S17. Electrochemical H_2O_2 reduction over the (a) CNTs, (b) O-CNTs, and (c) N,O-CNTs catalyst.

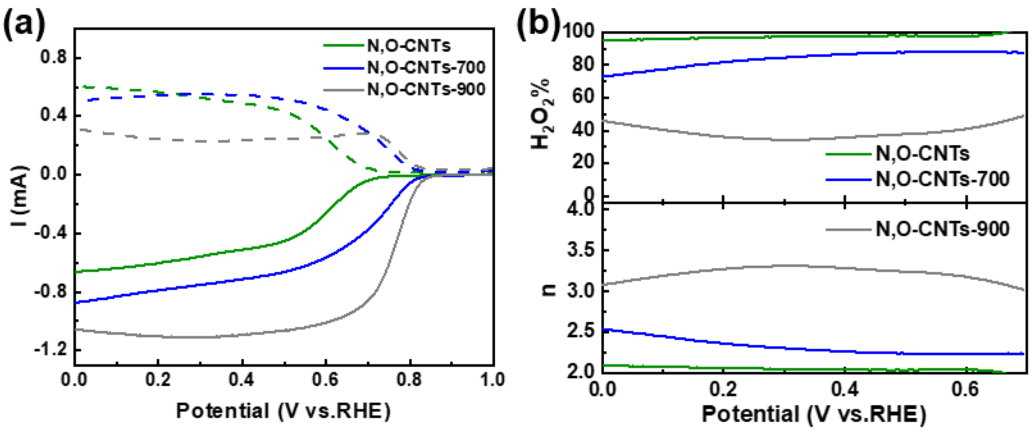

Figure S18. (a) RRDE voltammograms of different pyrolysis temperatures at 1600 rpm in O_2-saturated 0.1 M KOH electrolyte with the disc current, ring current adjusted by collection efficiency (Pt ring, disk area 0.283 cm^2, current is normalized with respect to disk geometric area). (b) Calculated H_2O_2 selectivity and electron transfer number during the potential sweep.

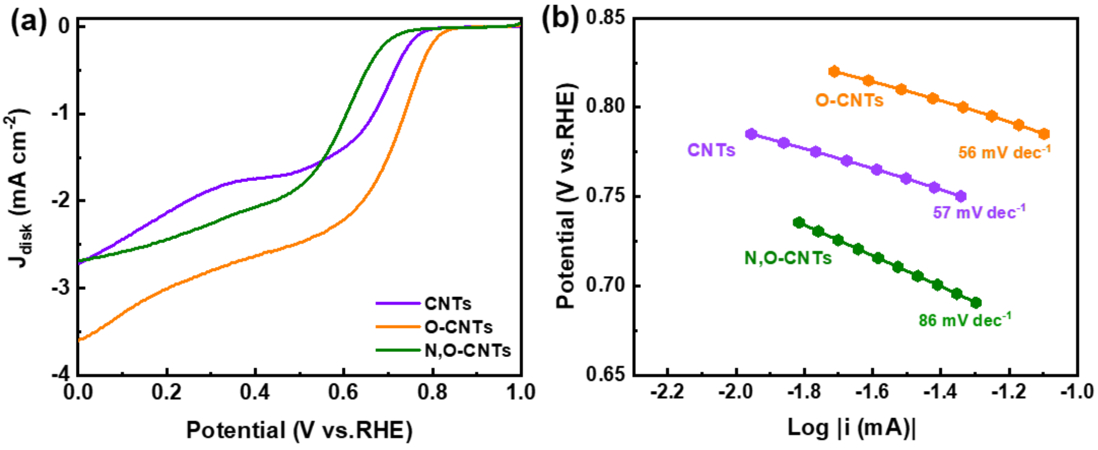

Figure S19. Disk current density (a) and Tafel plots (b) of CNTs, O-CNTs and N,O-CNTs.

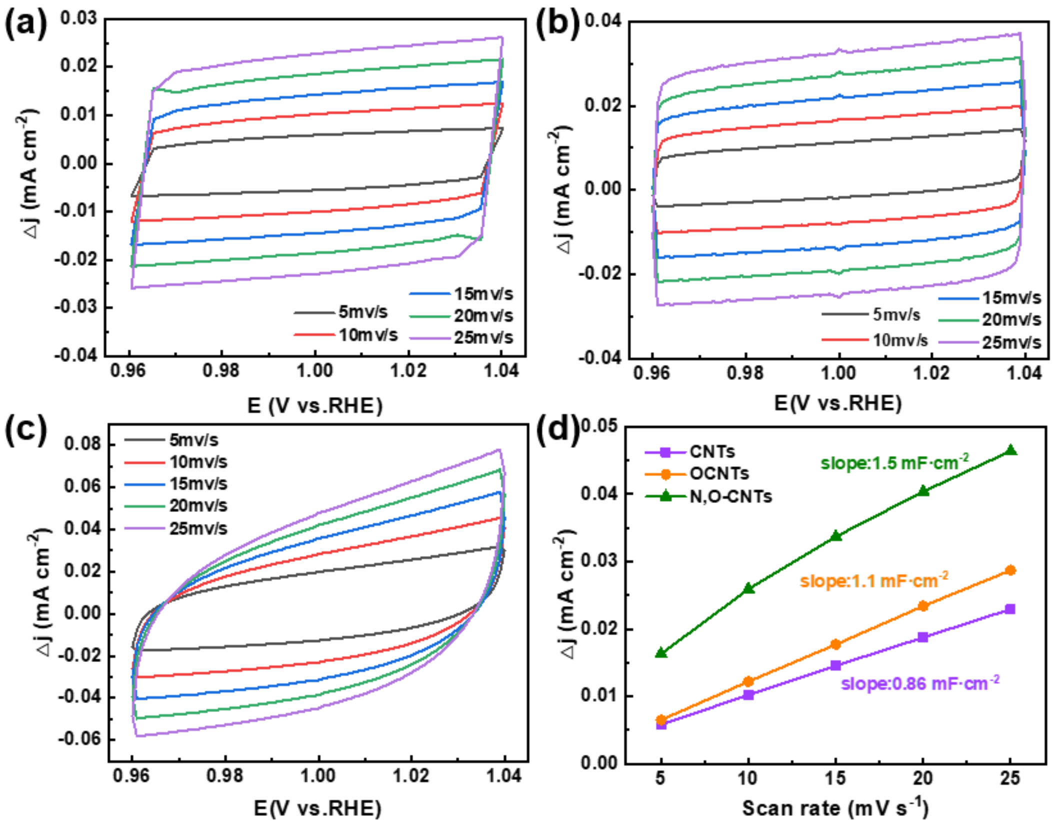

Figure S20. Cyclic voltammogram (CV) curves in the double layer region at scan rates of 5, 10, 15, 20, and 25 mV s^{-1} of (a) CNTs, (b)O-CNTs and (c) N,O-CNTs in 0.1 M KOH. (d) current density (taken at the potential of 1.00 V_{RHE}) as a function of scan rate derived from (a).

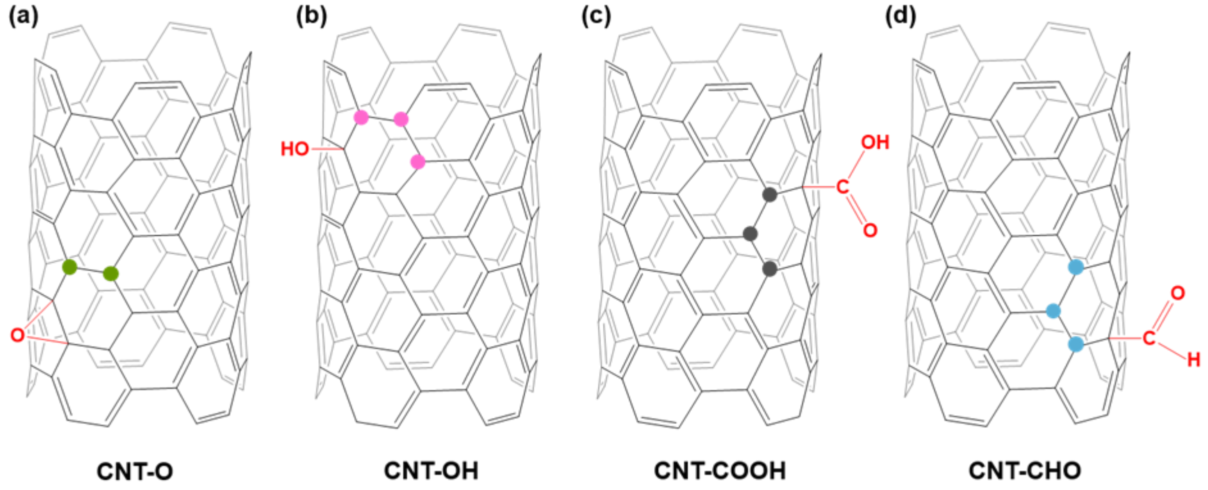

Figure S21. DFT calculation models of O-doping nanotube sites

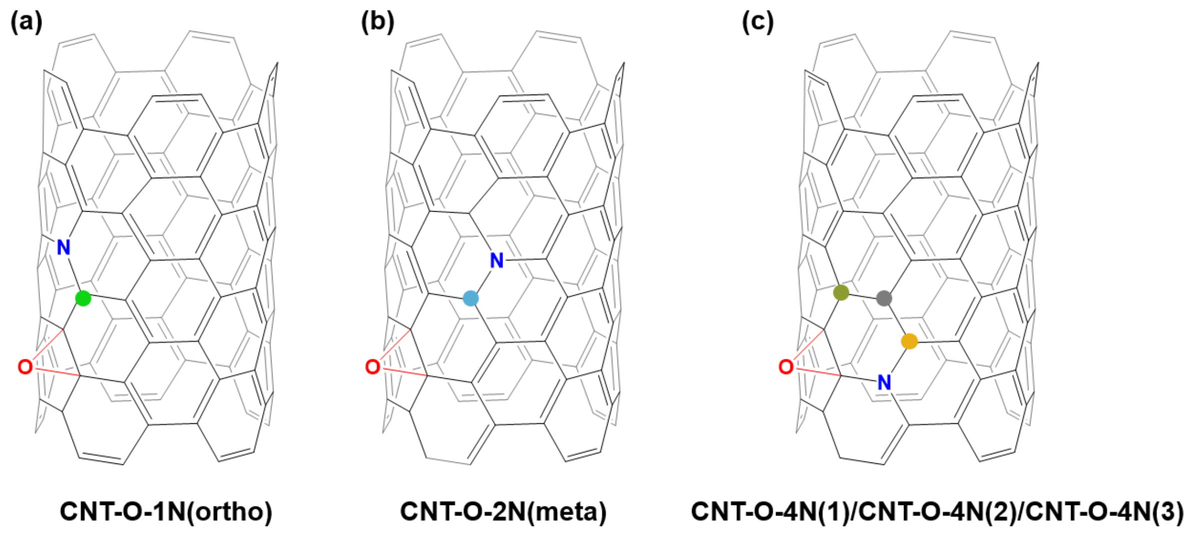

Figure S22. DFT calculation models of O and N co-doping carbon nanotube sites (CNT-O).

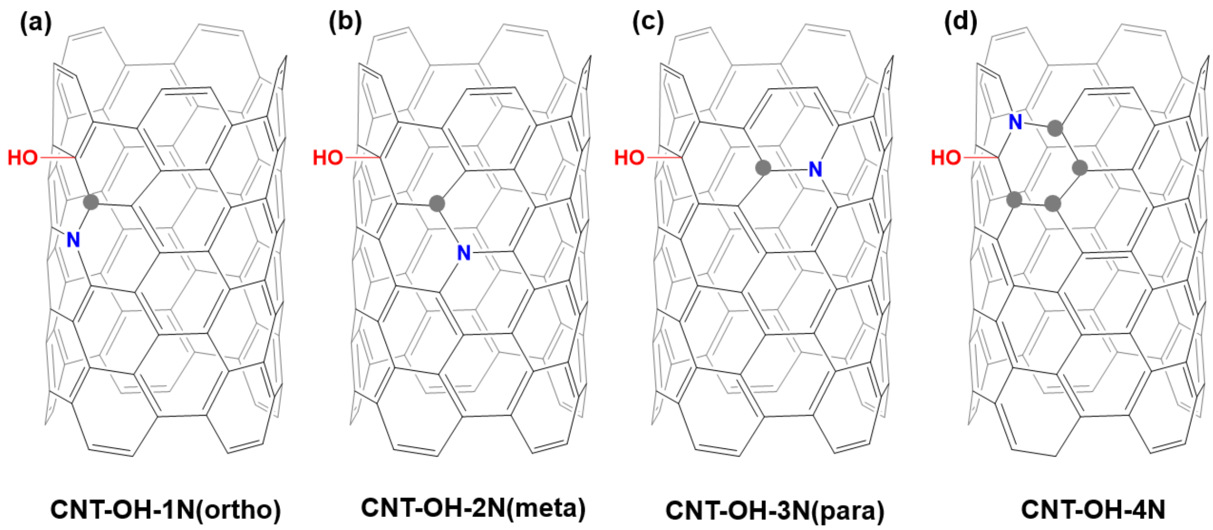

Figure S23. DFT calculation models of O and N co-doping carbon nanotube sites (CNT-OH).

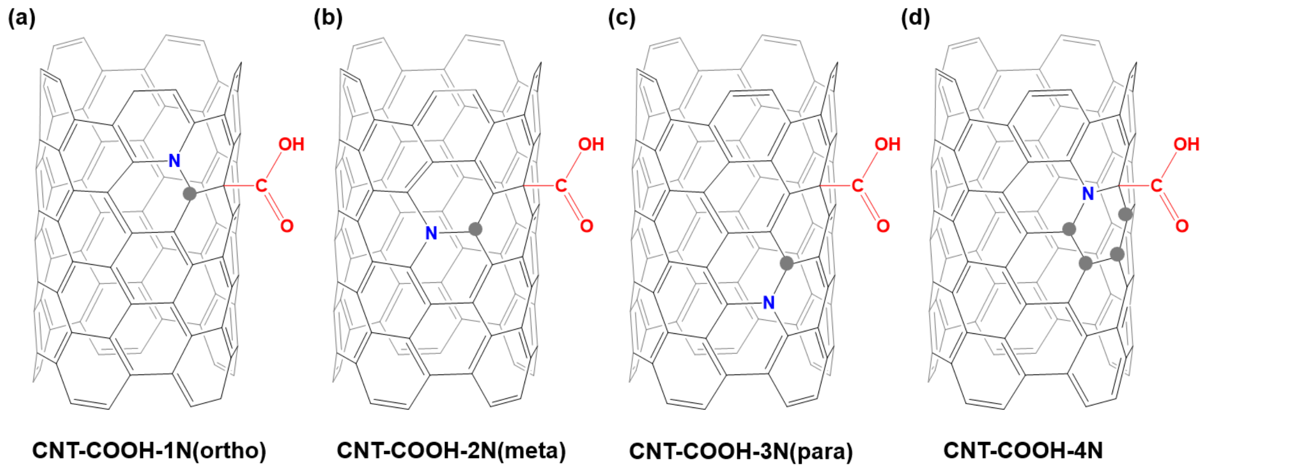

Figure S24. DFT calculation models of O and N co-doping carbon nanotube sites (CNT-COOH).

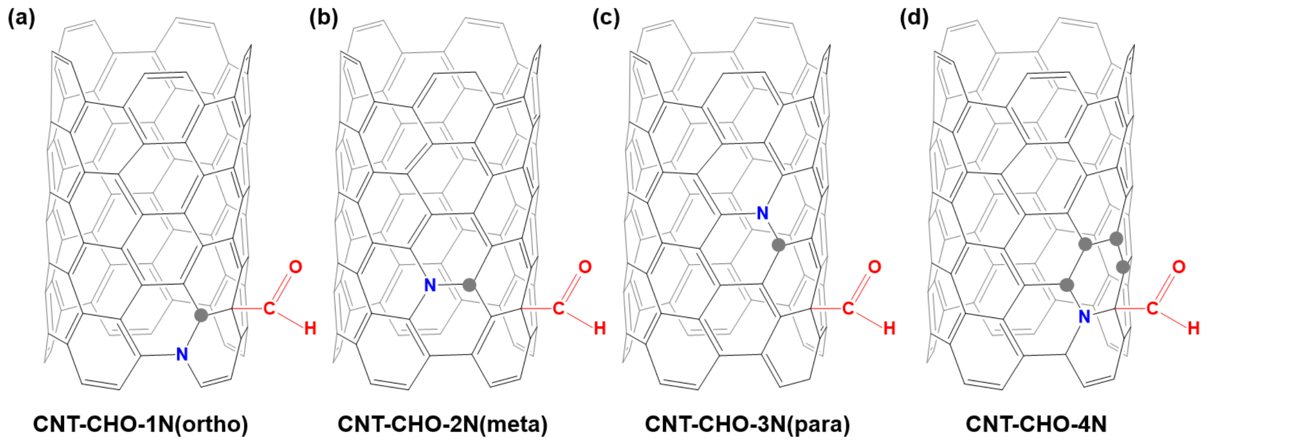

Figure S25. DFT calculation models of O and N co-doping carbon nanotube sites (CNT-CHO).

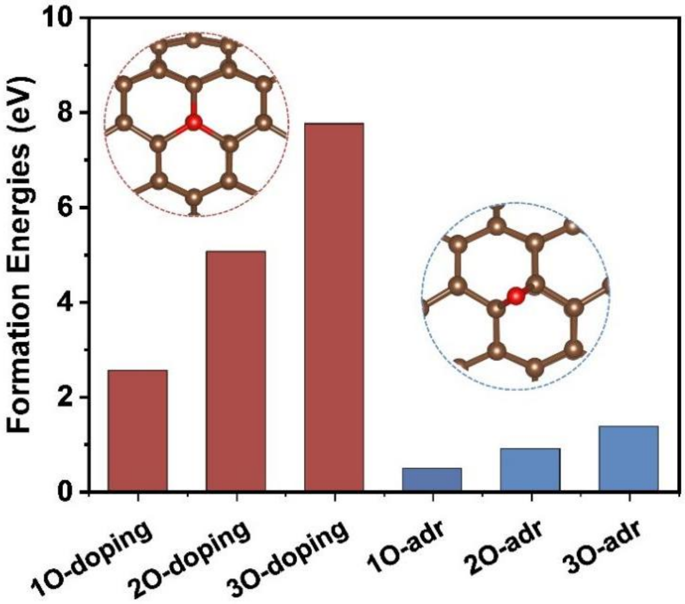

Figure S26. The formation energies of O-adsorbed and O-embedding CNT along increasing O atom number.

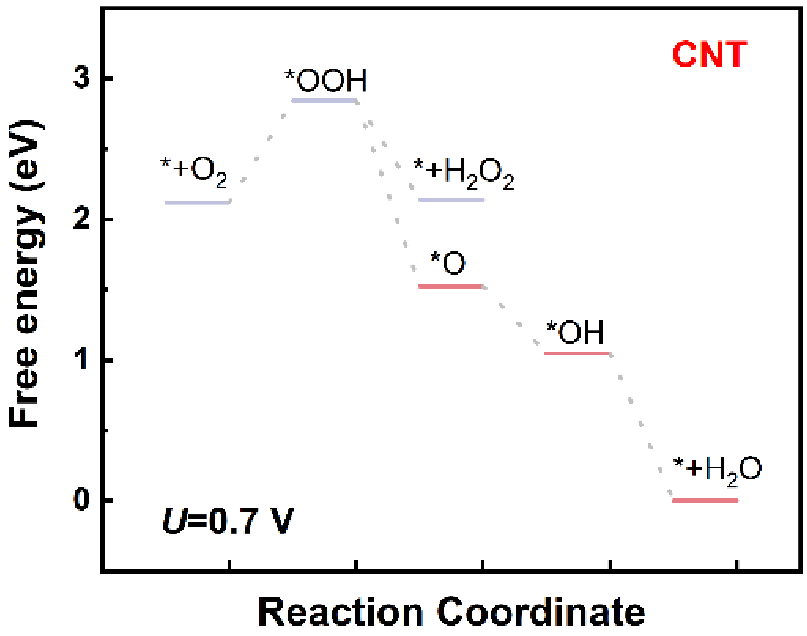

Figure S27. Free energy diagram of 2-electron/4-electron ORR on the original CNT site.

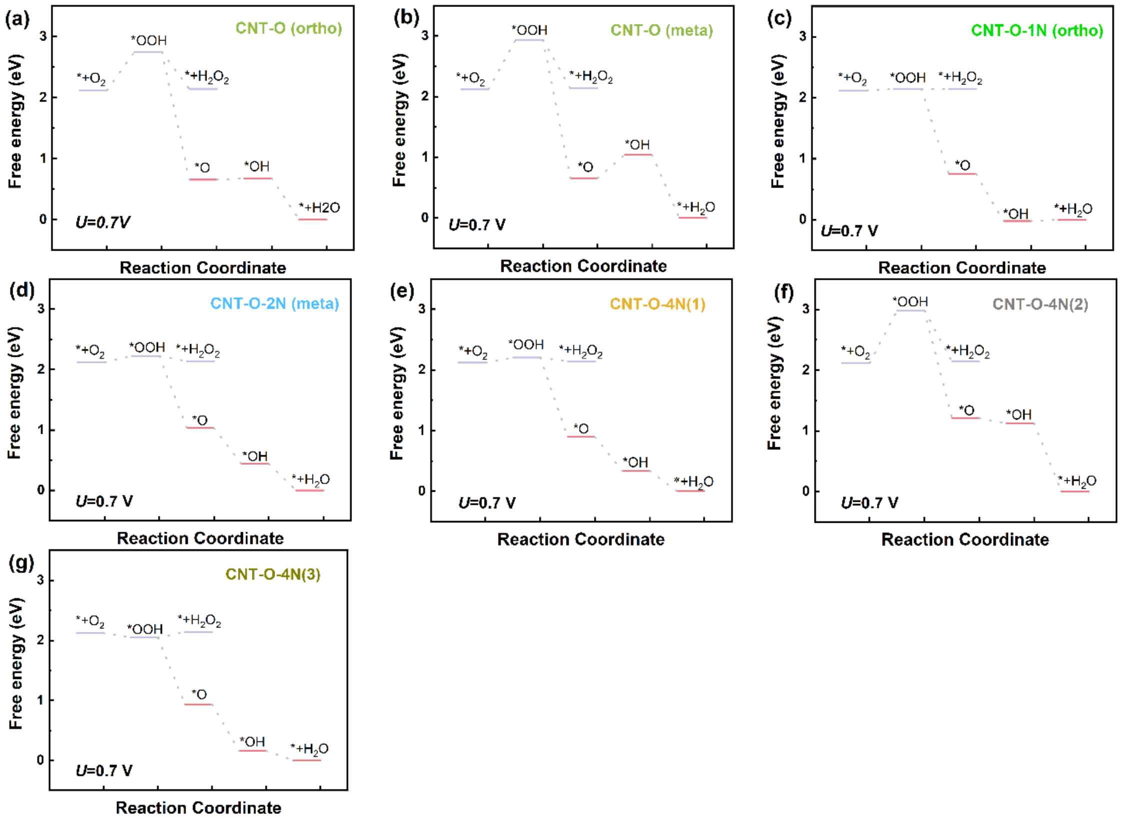

Figure S28. Free energy diagram of 2-electron/4-electron ORR on the original CNT-O and CNT-O, N site.

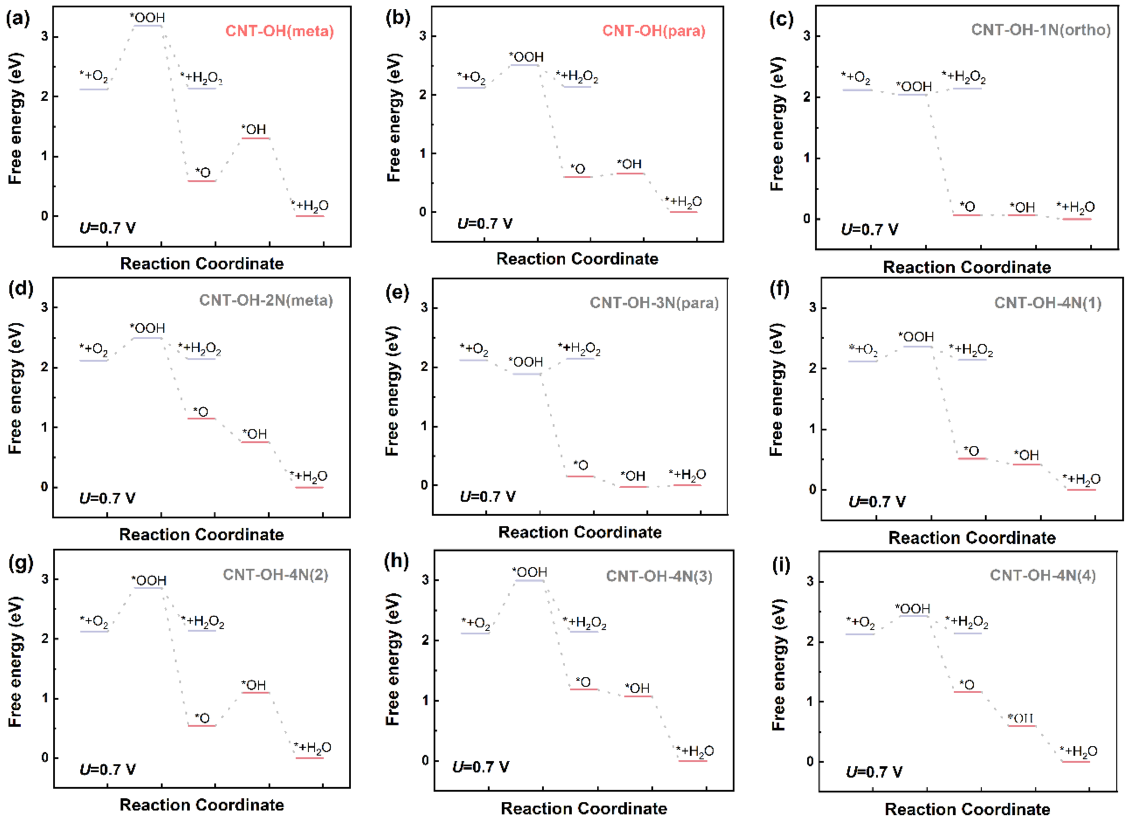

Figure S29. Free energy diagram of 2-electron/4-electron ORR on the original CNT-OH and CNT-OH, N site.

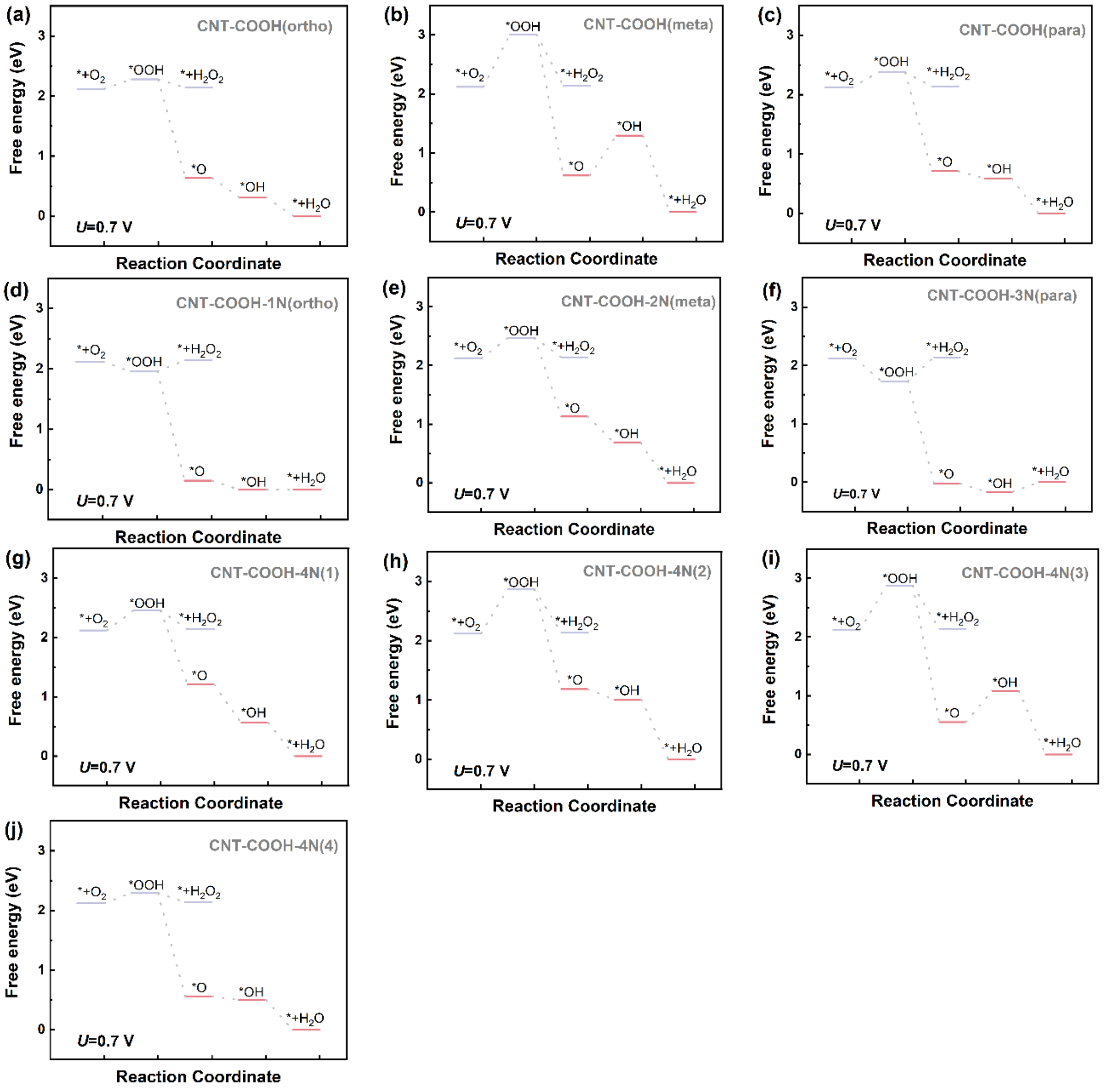

Figure S30. Free energy diagram of 2-electron/4-electron ORR on the original CNT-COOH and CNT-COOH, N site.

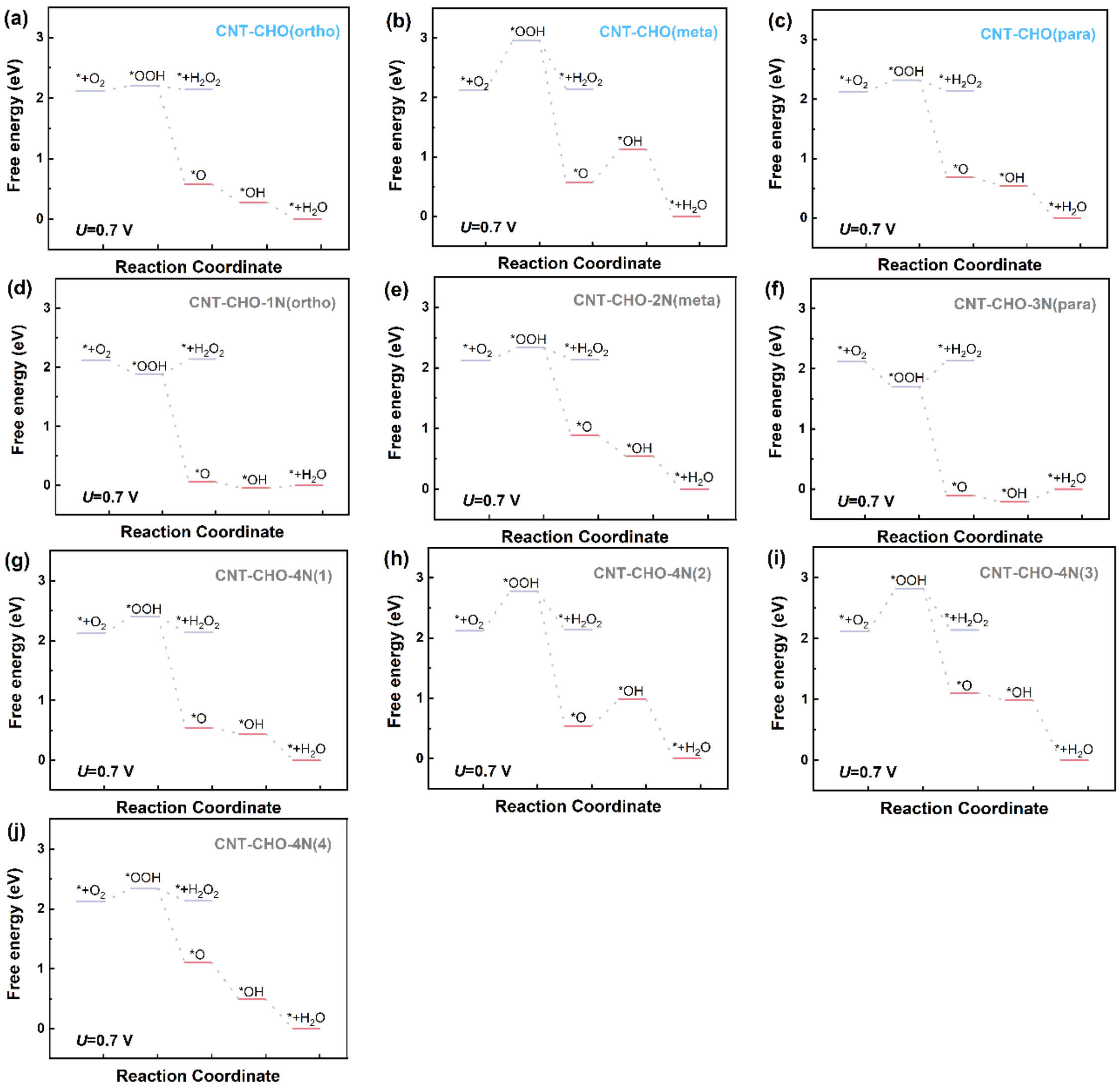

Figure S31. Free energy diagram of 2-electron/4-electron ORR on the original CNT-CHO and CNT-CHO, N site.

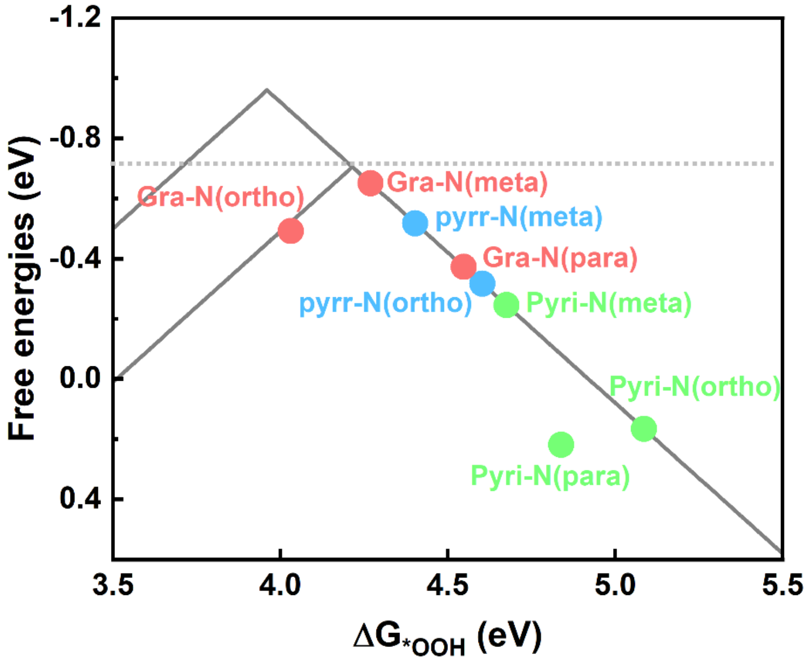

Figure S32. The volcano curve of N-doping CNT with pyrrole N and pyridine N.

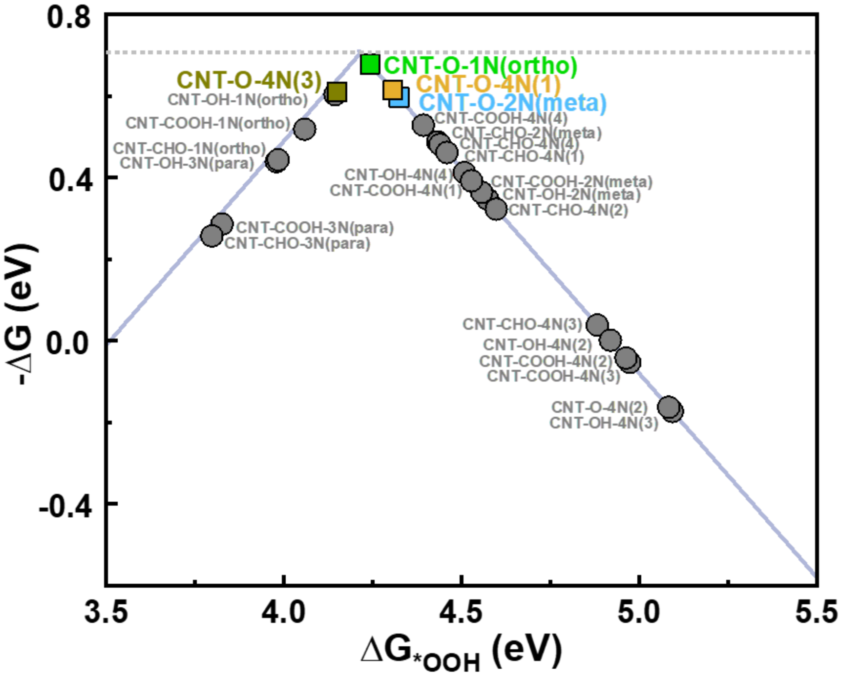

Figure S33. The ORR simulated activity volcano plot of all sites of N,O-CNTs.

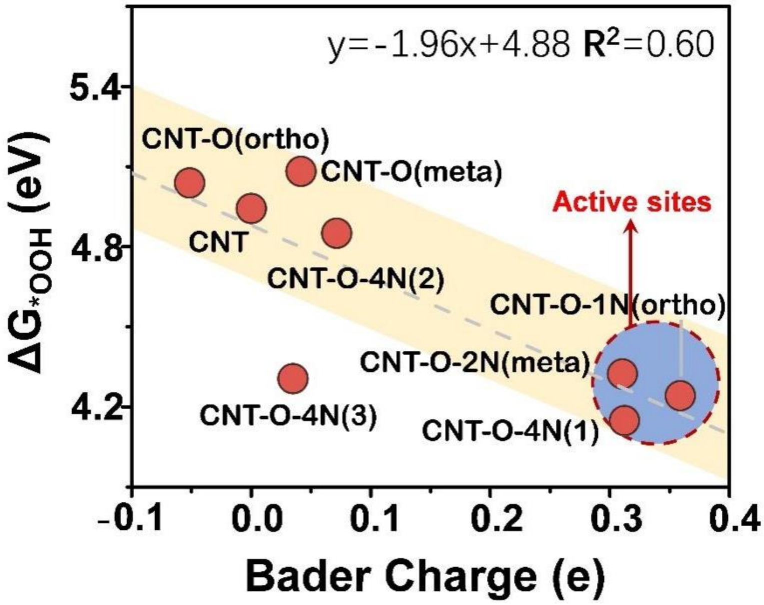

Figure S34. The linear relation of ΔG_{*OOH} and Bader charge of carbon sites.

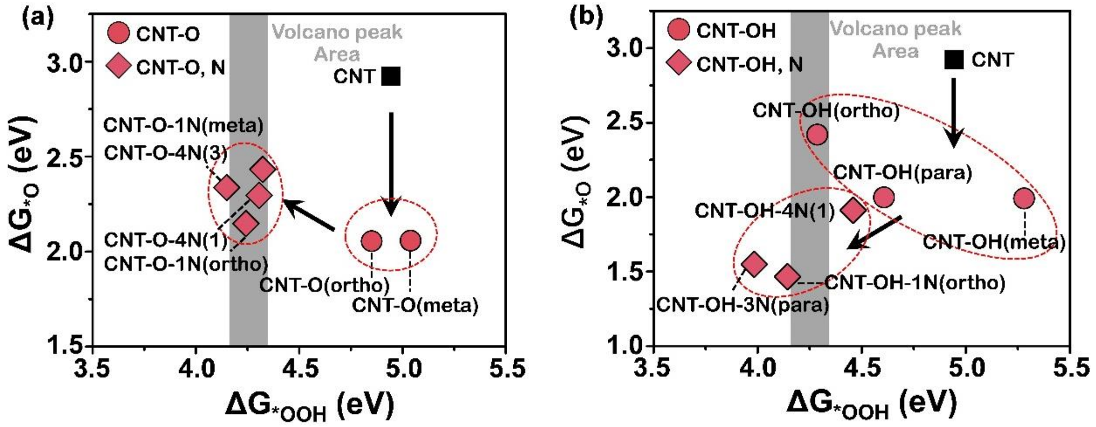

Figure S35. The distribution of ΔG_{*OOH} and ΔG_{*O} on (a) CNT, CNT-O, CNT-O, N and (b) CNT, CNT-OH, CNT-OH, N.

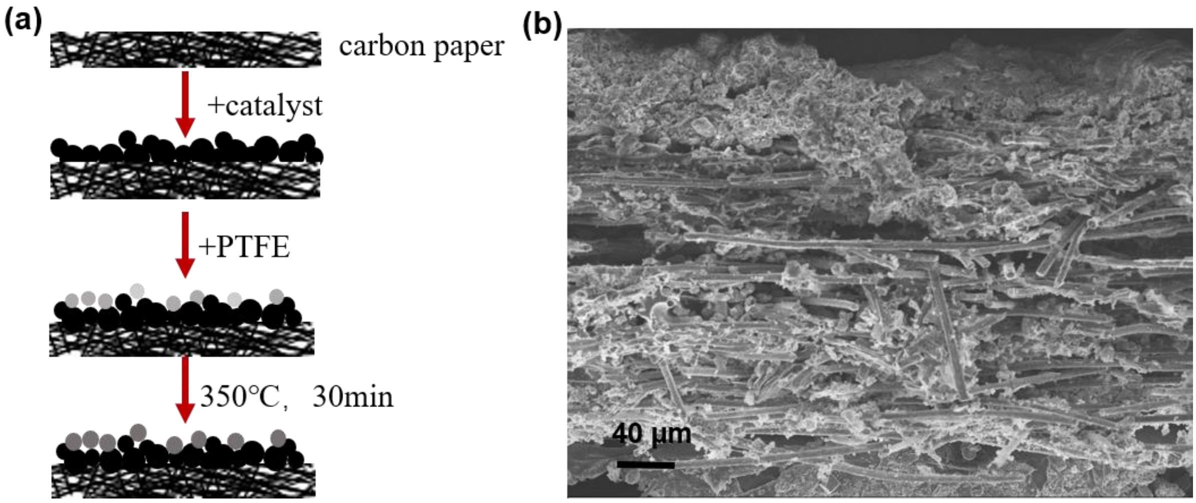

Figure S36. (a) The process of modifying carbon paper. (b) SEM images of the modified carbon paper substrate coated with N,O-CNTs.

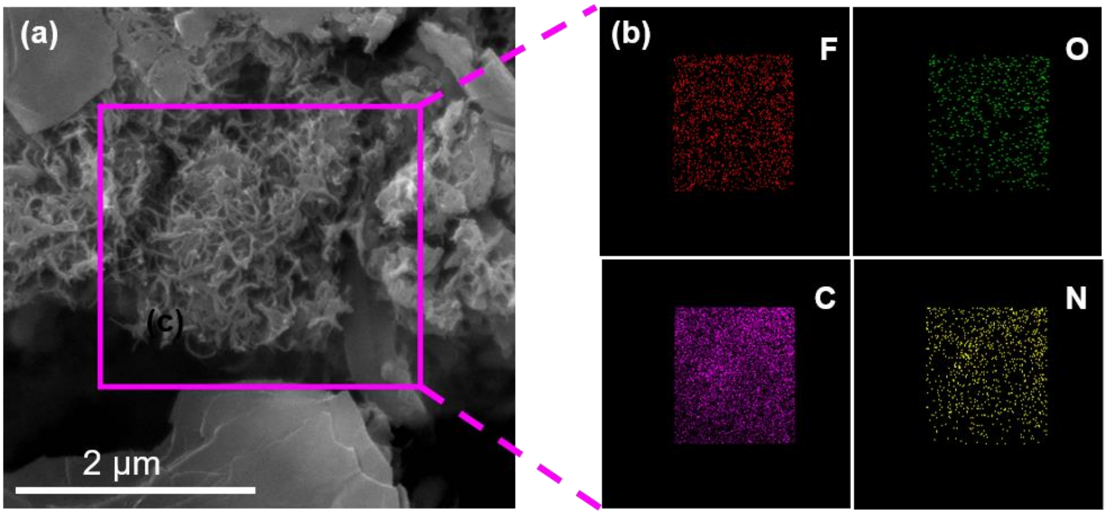

Figure S37. (a) SEM image of GDL supported catalyst and PTFE. Catalyst loading: 2 mg cm^{-2}, PTFE concentration: 5% wt, spray 1 mL on 1 cm^2 of carbon paper. (b) The corresponding elemental mapping of F (red), O (green), C (purple) and N (yellow) for the selected area in (a). The mapping of F elemental indicates the successful load of PTFE.

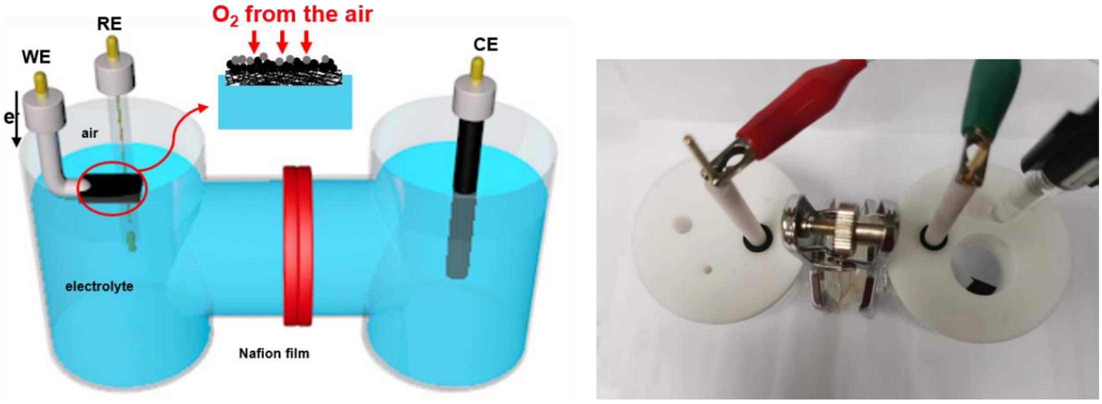

Figure S38. The electrochemical device used for the electrochemical synthesis of H_2O_2.

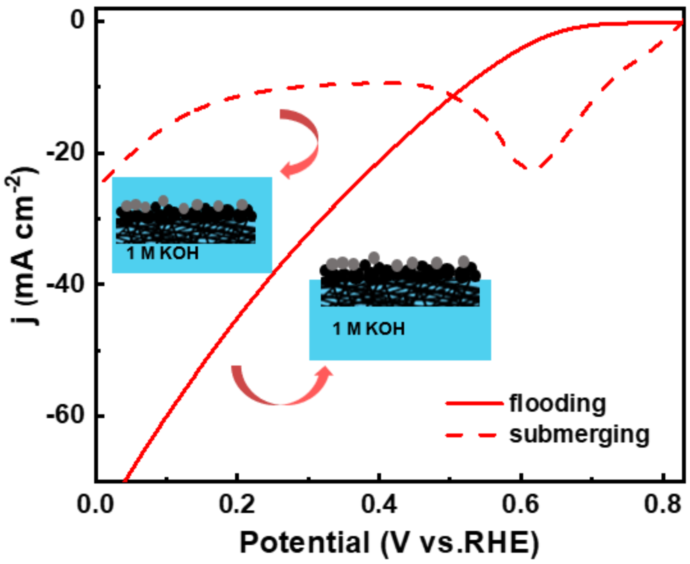

Figure S39. Polarization curves of electrode and the electrolyte at different relative positions.

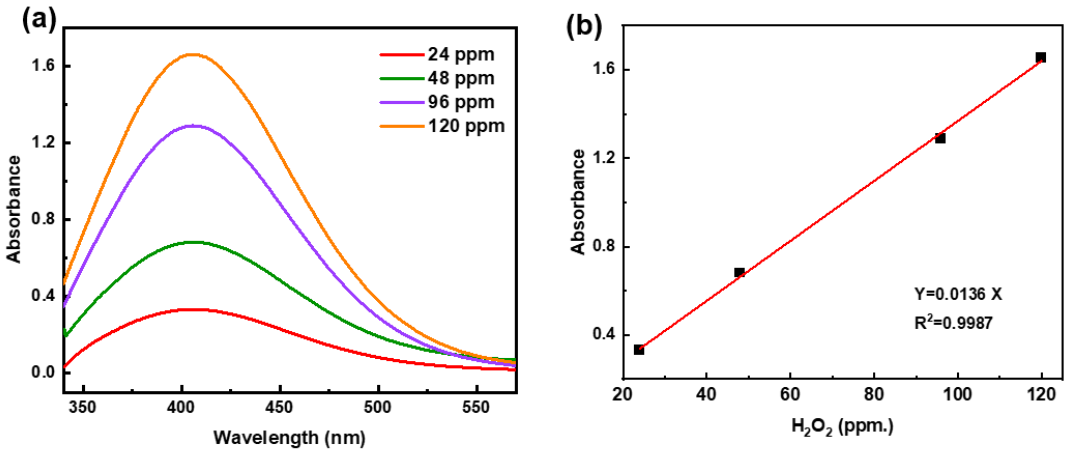

Figure S40. (a) UV-Vis absorption spectra for different concentration of H_2O_2 (1 mL) obtained in alkaline media. Titration solution (1 mL): 10 mM $Ti(SO_4)_2$ and 1 M H_2SO_4. (b) Relationship between the concentration of H_2O_2 and the absorbance of H_2TiO_4 at 408 nm in (a).

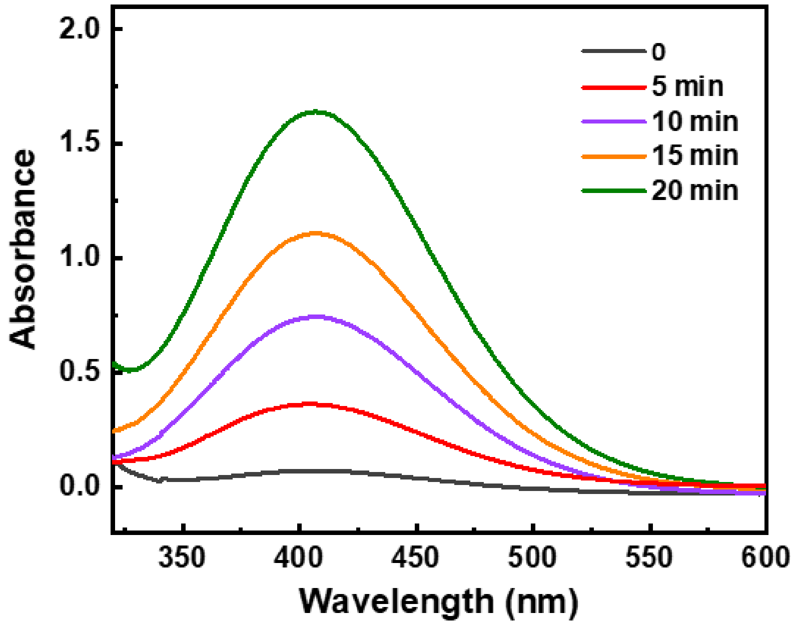

Figure S41. (a) UV-Vis absorption spectra of the generated H_2O_2 after different electrolytic times.

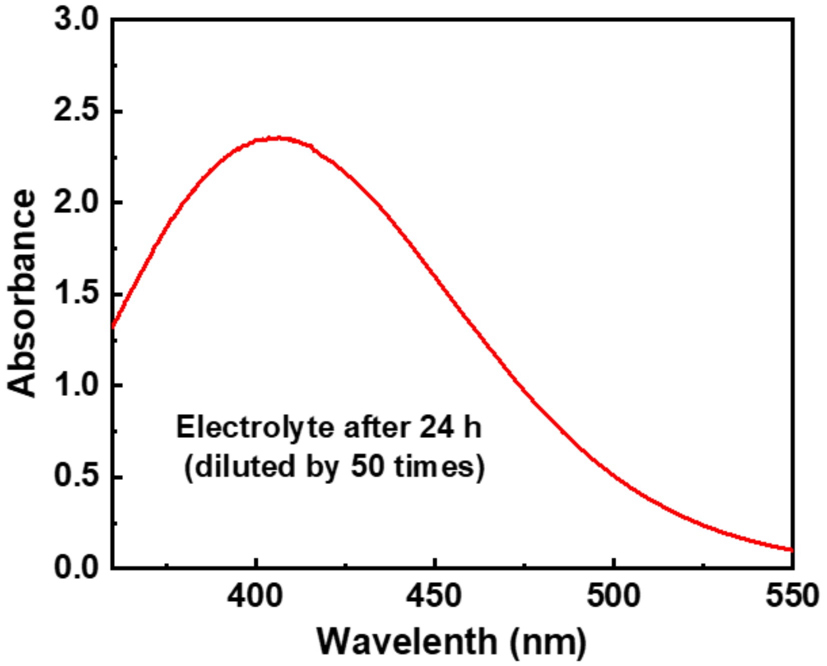

Figure S42. (a) UV-Vis absorption spectra of the generated H_2O_2 solution (diluted 50 times) of N,O-CNTs after 24 h of electrolysis.

4. Supplementary Tables

Table S1. Percentage concentrations of different oxygen species for OCNTs and N,O-CNTs detected by XPS .

sample	(C=O)%	(C-O-C)%	(C-OH)%
OCNTs	35	43	22
N,O-CNTs	28	48	24

Table S2. Relative contents of N,O-CNTs, N,O-CNTs-700 and N,O-CNTs-900 by XPS (at %) .

sample	C%	N%	O%
N,O-CNTs	74.02	21.59	4.39
N,O-CNTs-700	84.43	15.52	3.05
N,O-CNTs-900	94.89	2.62	2.49

Table S3. Comparing the performances of recently reported electrocatalysts for oxygen reduction to H_2O_2 in alkaline solution.

Journal	Catalysts	Potential range (V)	H_2O_2 %	Current density (mA cm^{-2})	Onset potential (V)	Tafel slope (mV dec^{-1})	H_2O_2 production rate	Ref
This work	N,O-CNTs	0-0.65	>95	-2.46@0.2V	~0.78	86	264.8 mmol g_{cat}^{-1} h^{-1} (50 mL, 1 cm^{-2})	
This work	O-CNTs	0.3-0.65	~80	-3.00@0.2V	~0.81	56	/	
This work	CNTs	0-0.65	~50	-2.13@0.2V	~0.79	57	/	
Nat. Commun.	B-C	0.4-0.7	~87	-3.00@0.3V	~0.77	/	7.36 mmol cm^{-2} h^{-1}	[5]
Nat. Catal.	O-CNTs	0.4-0.7	~89	-2.70@0.4V	~0.80	47	111.7 mmol g_{cat}^{-1} h^{-1}	[6]
Nat. Catal.	F-mrGO	/	>95	/	~0.78	/	/	[7]
Angew.Chem. Inter. Ed.	GOMC	0.2-0.6	~92	-2.46@0.2V	~0.77	48	51.25±4.37 mg L^{-1} h^{-1}	[8]
Angew.Chem. Inter. Ed.	HCNFs	0.2-0.7	~92	-2.88@0.2V	~0.85	75.6	6.37 mmol L^{-1} h^{-1}	[9]
▲Chem	g-N-CNHs	0.65-0.5	65-20	-1.10@0.3V	~0.71	70	73 mmol g_{cat}^{-1} h^{-1} cm^{-2}	[10]
▲ACS Catal.	oxo-G/NH_3 H_2O	0.1-0.6	81-83	-2.79@0.2V	~0.72	/	224.8 mmol g_{cat}^{-1} h^{-1}	[11]
J. Am. Chem. Soc.	BN-C1	0.2-0.8	60-90	-3.00@0.4V	~0.80	/	/	[12]
▲Carbon	N-MC-1	0.35-0.65	~69	-2.29@0.2V	~0.80	/	/	[13]
Chem. Commun.	cCTN	0.1-0.6	~83	-1.65@0.4V	~0.65	/	/	[14]
Nat. Mater.	Co1-NG(O)	0.1-0.8	~80	-2.80@0.2V	~0.80	/	418 mmol g_{cat}^{-1} h^{-1}	[15]
Nat. Mater.	Co1-NG(R)	0.1-0.8	50-20	-3.70@0.2V	~0.85	/	/	[15]
Nat. Commun.	O-C(Al)	0.4-0.65	~95	-2.82@0.4V	~0.82	52	867 mg^{-1} L^{-1} h^{-1}	[16]
Nat. Commun.	Fe-CNT	0.4-0.75	75-92	-3.3@0.4V	~0.81	/	461 mg^{-1} L^{-1} h^{-1}	[17]
Adv. Mater.	Co-POC-O	0.5-0.8	~85	-2.75@0.4V	~0.84	34	813 mg^{-1} L^{-1} h^{-1}	[18]
Adv. Mater.	Bi_2Te_3 NPs	0.2-0.6	≥95	-2.50@0.2V	~0.75	/	6.37 mmol L^{-1} h^{-1}	[19]
Small	$NiNb_2O_6$	0.2-0.6	80-96	1.70@0.2V	~0.72	100-120	996 mmol g_{cat}^{-1} h^{-1}	[20]

5. Supplementary References

[1] Q. Zhang, P. Liu, Z. Zhu, J. Zhang, F. Cao, *Corros. Sci.* **2020**, 164, 108312.

[2] a) G. Kresse, J. Furthmüller, *Comput. Mater. Sci.* **1996**, 6, 15; b) G. Kresse, J. Furthmüller, Y. Li, *Phys. Rev. B: Condens. Matter Mater. Phys* **2000**, 62, 11169.

[3] M. Ernzerhof, J. P. Perdew, *J. Chem. Phys.* **1998**, 109, 3313.

[4] J. K. Nørskov, J. Rossmeisl, A. Logadottir, L. Lindqvist, J. R. Kitchin, T. Bligaard, H. Jonsson, *J. Phys. Chem. B* **2004**, 108, 17886.

[5] Y. Xia, X. Zhao, C. Xia, Z.-Y. Wu, P. Zhu, J. Y. T. Kim, X. Bai, G. Gao, Y. Hu, J. Zhong, Y. Liu, H. Wang, *Nat. Commun.* **2021**, 12, 4225.

[6] Z. Lu, G. Chen, S. Siahrostami, Z. Chen, K. Liu, J. Xie, L. Liao, T. Wu, D. Lin, Y. Liu, *Nat. Catal.* **2018**, 1, 156.

[7] H. W. Kim, M. B. Ross, N. Kornienko, L. Zhang, J. Guo, P. Yang, B. D. McCloskey, *Nat. Catal*. **2018**, 1, 282.

[8] Y. J. Sa, J. H. Kim, S. H. Joo, *Angew. Chem., Int. Ed*. **2019**, 58, 1100.

[9] K. Dong, J. Liang, Y. Wang, Z. Xu, Q. Liu, Y. Luo, T. Li, L. Li, X. Shi, A. M. Asiri, *Angew. Chem., Int. Ed.* **2021**, 133, 10677.

[10] D. Iglesias, A. Giuliani, M. Melchionna, S. Marchesan, A. Criado, L. Nasi, M. Bevilacqua, C. Tavagnacco, F. Vizza, M. Prato, P. Fornasiero, *Chem* **2018**, 4, 106.

[11] L. Han, Y. Sun, S. Li, C. Cheng, C. E. Halbig, P. Feicht, J. L. Hübner, P. Strasser, S. Eigler, *ACS Catal.* **2019**, 9, 1283.

[12] S. Chen, Z. Chen, S. Siahrostami, D. Higgins, D. Nordlund, D. Sokaras, T. R. Kim, Y. Liu, X. Yan, E. Nilsson, *J. Am. Chem. Soc.* **2018**, 140, 7851.

[13] V. Perazzolo, C. Durante, R. Pilot, A. Paduano, J. Zheng, G. A. Rizzi, A. Martucci, G. Granozzi, A. Gennaro, *Carbon* **2015**, 95, 949.

[14] L.-Z. Peng, P. Liu, Q.-Q. Cheng, W.-J. Hu, Y. A. Liu, J.-S. Li, B. Jiang, X.-S. Jia, H. Yang, K. Wen, *Chem. Commun.* **2018**, 54, 4433.

[15] E. Jung, H. Shin, B.-H. Lee, V. Efremov, S. Lee, H. S. Lee, J. Kim, W. H. Antink, S. Park, K.-S. Lee, *Nat. Mater.* **2020**, 19, 436.

[16] Q. Yang, W. Xu, S. Gong, G. Zheng, Z. Tian, Y. Wen, L. Peng, L. Zhang, Z. Lu, L. Chen, *Nat. Commun.* **2020**, 11, 5478.

[17] K. Jiang, S. Back, A. J. Akey, C. Xia, Y. Hu, W. Liang, D. Schaak, E. Stavitski, J. K. Nørskov, S. Siahrostami, H. Wang, *Nat. Commun.* **2019**, 10, 3997.

[18] B. Q. Li, C. X. Zhao, J. N. Liu, Q. Zhang, *Adv. Mater.* **2019**, 31, 1808173.

[19] N. Zhang, F. Zheng, B. Huang, Y. Ji, Q. Shao, Y. Li, X. Xiao, X. Huang, *Adv. Mater.* **2020**, 32, 1906477.

[20] C. Liu, H. Li, J. Chen, Z. Yu, Q. Ru, S. Li, G. Henkelman, L. Wei, Y. Chen, *Small* **2021**, 17, 2007249.
